# A Web-Based Resilience-Enhancing Program to Improve Resilience, Physical Activity, and Well-being in Geriatric Population: Randomized Controlled Trial

**DOI:** 10.2196/53450

**Published:** 2024-07-25

**Authors:** Yi-Chen Wu, Shu-Fen Shen, Liang-Kung Chen, Heng-Hsin Tung

**Affiliations:** 1 College of Nursing and Health Sciences, Dayeh University Changhua Taiwan; 2 Department of Nursing, Mackay Medical College Taipei Taiwan; 3 Center for Geriatrics and Gerontology Taipei Veterans General Hospital Taipei Taiwan; 4 Center for Healthy Longevity and Aging Sciences National Yang Ming Chiao Tung University Taipei Taiwan; 5 Taipei Municipal Gan-Dau Hospital Taipei Taiwan; 6 Department of Nursing,National Yang Ming Chiao Tung University Taipei Taiwan

**Keywords:** geriatric population, community-dwelling older adult, web-based resilience-enhancing program, resilience, physical activity, well-being, pandemic.

## Abstract

**Background:**

Resilience is a protective factor in healthy aging, helping to maintain and recover physical and mental functions. The Resilience in Illness Model has proven effective in fostering resilience and well-being. Physical activity is crucial for older adults’ independence and well-being, even as aging causes a progressive decline. Additionally, older adults face challenges such as spousal loss and physical disability, making preventive intervention strategies necessary.

**Objective:**

This study aims to develop and evaluate a web-based program to enhance resilience, physical activity, and well-being among community-dwelling older adults. Additionally, we aim to gather feedback on the program’s strengths and limitations.

**Methods:**

A 4-week resilience-enhancing program was created, incorporating role-play and talk-in-interaction and focusing on 3 key skills: coping, control belief, and manageability. The program included scenarios such as becoming widowed and suffering a stroke, designed to engage older adults. A pilot test preceded the intervention. As a result of the COVID-19 pandemic, the program shifted from in-person to web-based sessions. A single-blind, parallel-group, randomized controlled trial was conducted. Participants aged over 65 years were recruited offline and randomly assigned to either an intervention or control group. A certified resilience practitioner delivered the program. Outcomes in resilience, physical activity, and well-being were self-assessed at baseline (T0), 4 weeks (T1), and 12 weeks (T2) after the program. A mixed methods approach was used to evaluate feedback.

**Results:**

A web-based participatory program enhancing 3 skills—coping, control belief, and manageability for resilience—was well developed. Among 96 participants, 63 were randomized into the intervention group (n=31) and the control group (n=32). The mean age in the intervention group was 69.27 (SD 3.08) years and 74.84 (SD 6.23) years in the control group. Significant between-group differences at baseline were found in age (t45.6=–4.53, *P*<.001) and physical activity at baseline (t61=2.92, *P*=.005). No statistically significant between-group differences over time were observed in resilience (SE 7.49, 95% CI –10.74 to 18.61, *P*=.60), physical activity (SE 15.18, 95% CI –24.74 to 34.74, *P*=.74), and well-being (SE 3.74, 95% CI –2.68 to 11.98, *P*=.21) after controlling for baseline differences. The dropout rate was lower in the intervention group (2/31, 6%) compared with the control group (5/32, 16%). Moreover, 77% (24/31) of participants in the intervention group completed the entire program. Program feedback from the participants indicated high satisfaction with the web-based format and mentorship support.

**Conclusions:**

This study demonstrated that a web-based resilience-enhancing program is appropriate, acceptable, feasible, and engaging for community-dwelling older adults. The program garnered enthusiasm for its potential to optimize resilience, physical activity, and well-being, with mentorship playing a crucial role in its success. Future studies should aim to refine program content, engagement, and delivery methods to effectively promote healthy aging in this population.

**Trial Registration:**

ClinicalTrials.gov NCT05808491; https://clinicaltrials.gov/ct2/show/NCT05808491

## Introduction

Population aging is a pressing global issue today, and the World Health Organization (WHO) advocates preserving intrinsic capacity and functional ability to promote healthy aging [1]. Resilience has been defined by the National Institutes of Health as a positive health outcome [2], and it remains a significant research focus aimed at facilitating healthy aging for both individuals and society at large [3,4]. Resilience is individually described as a protective factor—the capacity to overcome adversity and maintain or regain well-being [5,6]. It enables individuals to bounce back from setbacks by adapting quickly to unfavorable situations, which typically fluctuate across one’s lifespan. Moreover, high resilience later in life is associated with optimal health and minimal disability [7,8], as noted even during the COVID-19 pandemic [9]. Importantly, accumulating evidence has shown that resilience interventions have produced positive effects and enhanced well-being in various populations, including the geriatric population [10-14]. However, intervention programs to promote well-being among Taiwanese community-dwelling older adults have yielded inconclusive results. For instance, a reexperiencing gratitude intervention improved well-being through the mediated effect of grateful emotions [15], whereas mental health promotion programs and reminiscence group activities enhanced well-being among institutionalized older adults [16,17].

The Resilience in Illness Model (RIM) has been shown to cultivate resilience and well-being [18,19]. Developed by Haase et al [20], this model adopts a positive health perspective to evaluate the combined contributions of biological, behavioral, and psychosocial factors in adolescents and young adults with chronic illnesses. In the RIM, there are 2 risk factors, namely, illness-related risk and defensive coping, along with 5 protective factors, including spiritual perspective, social integration, hope-derived meaning, family environment, and courageous coping. The model identifies 3 outcomes: resilience resolution, self-transcendence, and a sense of well-being [21]. According to the RIM, stressors, such as life events or disease-related health conditions, positively affect defensive coping and negatively impact courageous coping. Protective factors such as courageous coping (which includes confrontive, optimistic, and supportive strategies) and hope-derived meaning are associated with enhanced resilience [20]. Furthermore, coping, defined as behaviors that protect oneself by avoiding psychological harm from negative experiences, along with control belief, the ability to manage unexpected situations and facilitate cognitive strategies to lessen the negative consequences of adversity, and manageability, the ability to access sufficient resources to deal with adversity, are 3 key components in enhancing resilience [22-25].

Advanced age brings progressive structural and functional deterioration of physiological systems, negatively impacting an individual’s daily life abilities. Consequently, older adults gradually become more dependent on support and assistance from others [26]. Physical functionality in older adults is determined by their ability to perform daily activities, and adequate physical activity is essential for maintaining or enhancing this functionality [27]. A high level of physical activity has been well-documented in numerous studies to provide significant and beneficial effects on older adults’ independence, quality of life, and well-being [28-32]. In the context of healthy aging, physical function is positively associated with resilience. Additionally, physical activity influences the relationship between resilience and mental health [32,33].

In late adulthood, spousal loss is considered a significant stressor, often the most overwhelming transition, profoundly impacting both mental and physical well-being [34]. Spousal bereavement compromises older adults’ well-being, often accompanied by other strains and losses such as compromised physical mobility, health, and sensory functions (eg, vision and hearing). Psychologically, it disrupts the meaning and order of daily life. The psychological adjustment in older bereaved spouses varies widely, influenced by protective factors such as resilience and personal and social resources, including social support, personality, and prior mental health [35]. Another adverse health outcome related to aging is the increased risk of chronic diseases, which contributes to higher disability incidence and functional decline among older adults. These outcomes result in illness, disability, death, and increased health care costs for individuals and nations [36]. The development of symptomatology and progression in chronic diseases are influenced by psychological processes such as stress and resilience [37]. Additionally, the prevalence of chronic illness among Taiwanese older adults aged over 65 years has reached nearly 90% [38].

COVID-19 rapidly spread worldwide from December 2019 and caused a traumatic impact on the global economy, education, hospitality, sports, leisure, and especially individual daily life [39]. Older adults were considered the most vulnerable group during the pandemic, experiencing unintended physical, mental, emotional, social, and financial consequences due to COVID-19 control strategies [40]. Moreover, studies during the pandemic showed a significant decrease in physical activity among older adults, while those who regularly engaged in moderate or vigorous physical activity demonstrated higher resilience [30,40-42]. These studies also highlighted that resilience plays a protective and buffering role in older adults’ psychological, physical, social, and economic health dimensions during the recovery process from the COVID-19 pandemic [43].

Healthy aging, which promotes well-being in older age by enhancing resilience, is the process of developing and maintaining independence, purpose, vitality, and quality of life in the geriatric population despite facing unexpected adversity [29,44]. This longevity should ideally correspond to an increase in healthy, disability-free years of life [45,46]. Population aging increases the burden on health care systems globally [45]. Health services play a pivotal role in healthy aging [46], using well-designed strategies to address the specific needs of older adults. It is crucial to develop preventive intervention strategies for the aging population to mitigate adverse aging-related health outcomes. The purpose of this study was to develop a web-based resilience-enhancing program for the geriatric population, evaluate its efficacy, and gather feedback on its strengths and limitations. The research hypothesis was that the web-based resilience-enhancing program improved resilience, physical activity, and well-being in community-dwelling older adults.

## Methods

### Study Design and Setting

The study was a 1:1 parallel-group randomized controlled trial (RCT) using purposive sampling to recruit community-dwelling older adults. Participant selection was fully automated and randomized equally to either the intervention or control group. As a result of social distancing and quarantine during the COVID-19 pandemic, we substituted the in-person 4-week resilience-enhancing program with a web-based approach. Outcomes, including resilience, physical activity, and well-being, were measured at baseline (T0), 4 weeks (T1), and 12 weeks (T2) after completing the resilience program or after allocation.

### Recruitment

We invited community-dwelling older adults aged 65 years or older, willing to participate, and able to communicate in Mandarin or another Chinese dialect in Northern Taiwan. Participants with clinically significant severe cognitive dysfunction or psychotic disorders, or those currently undergoing treatment for progressive malignant neoplasms as self-reported, were excluded. We conducted a simple recruitment briefing at a community center, where researchers used posters to promote the study. Community-dwelling older adults interested in participating received study information and a consent form individually. Upon providing consent, participants completed demographic information and a baseline survey either independently or with assistance from researchers.

### Randomization and Blinding

A single-blind RCT was conducted in 2 communities, comparing a web-based resilience-enhancing program with standard clinical practice. Participants were not blinded to allocation due to the nature of the training. Eligible participants were randomized in a 1:1 ratio to either the intervention or control group using a computer-generated random assignment scheme. Block randomization with block sizes of 4 was used to maintain balance throughout the trial. An independent third party managed blinding of the characteristic study participants. The researchers involved in data analysis were blinded to the group allocation.

### Intervention

The web-based resilience-enhancing program was designed with consideration for the challenges faced by older adults in later life. It consists of 3 phases that include designing and testing role-playing scenarios. The first author (YCW) completed a training program and obtained certification as a resilience practitioner before designing the study.

The in-depth training program for resilience practitioners was provided by Dr Chris Johnstone, a leading resilience trainer in the United Kingdom and author of “Seven Ways to Build Resilience: Strengthening Your Ability to Deal with Difficult Times.” The program included evidence-based resilience tools, strategies, strengths, resources, and insights tailored for health care providers in roles supporting resilience among others dealing with stress and adversity. The toolkits delivered in the resilience practitioner training program included storyboarding, emotional first aid, flexible thinking, overload management, problem-solving strategies, support strengthening, and stickability. This training course is accredited by the Association for Coaching for continuing professional development (see Multimedia Appendix 1).

Coping, control belief, and manageability were the 3 key skills in the resilience-enhancing program we developed. Coping skills were demonstrated through emotional first aid in the practitioner training program, utilizing self-compassion, mindfulness, and acceptance and commitment therapy. Control belief skills were demonstrated through flexible thinking in the practitioner training program, incorporating cognitive therapy and thinking skills taught in the Penn Resilience Program. Manageability skills were demonstrated through overload management and problem-solving strategies in the practitioner training program. All therapies and strategies used in our resilience-enhancing program have been shown to bolster resilience in the general population [6,47-49].

### Design Phase

**Figure 1:**
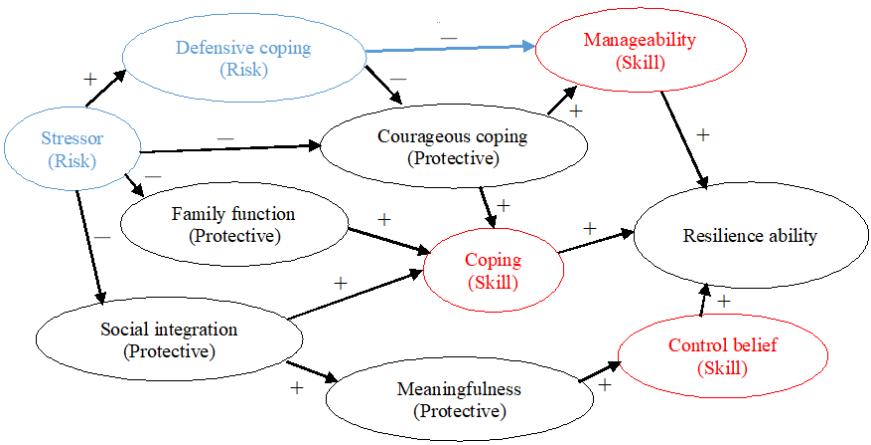
Modified RIM showed a stressor affected defensive coping positively and affected three protective factors as courageous coping, family function and social integration negatively, whereas resilience ability, as an outcome, was enhanced by the three skills directly and other protective factors, such as courageous coping, family function, social integration, meaningfulness indirectly. Blue circles indicate risk factors, whereas red circles indicate the three skills were practiced in the program. Black circles indicate protective factors and outcome. The arrows represent in direction of interaction between two circles. The plus sign + indicates positive effects, while the minus sign－indicate negative effects.

The resilience-enhancing program in this study was designed based on Haase et al’s [14,18-20] RIM and related research. We identified stressors in older adulthood as including life events (eg, spousal loss) and disease-related health conditions (eg, suffering from paralysis after a stroke). Additionally, 3 main resilience skills—coping, control belief, and manageability—were incorporated into the modified RIM (Figure 1).

Furthermore, the learning approach we applied was situated teaching, which involves learning by doing and applying knowledge to real-life situations that older adults can relate to. Situated teaching is a social process that encourages the coconstruction and internalization of knowledge [50]. Methods used to apply situated learning included group activities, role-plays, scenario-based learning, and the use of technology, providing individuals with opportunities to practice new knowledge and skills in semirealistic contexts rather than simply reading a manual. This approach situates learning within specific social and physical environments [50,51].

In the design phase, we developed a participatory program to enhance 3 skills—coping, control belief, and manageability—for community-dwelling older adults based on a modified version of the RIM. Two scenarios were created based on the needs of community-dwelling older adults: a stressful life event of becoming widowed and a disease-related event of suffering from stroke, designed to engage participants in learning. The participatory program included 2 instructional scenarios, missions, developed scripts, and feedback for participants in 2 events, followed by resilient factor assessments to identify individual protective and risk factors (see Multimedia Appendix 2). Additionally, independent preparatory learning included 2 short films introducing resilience and demonstrating resilience skills, which were produced and provided via YouTube (Alphabet Inc./Google LLC).

### Intervention Phase

The resilience-enhancing program involved weekly 60-minute role-playing and interactive talk sessions over 4 weeks. It comprised 4 sections: understanding resilience through e-learning, practicing resilience skills with a stressful life event and a disease-related event, and evaluation. We implemented a blended synchronous and asynchronous learning approach, which included independent preparatory learning with 2 microfilms (see Multimedia Appendix 3) on YouTube before 3 small-group facilitated sessions on weekly modules led by a certified resilience practitioner. Following a pilot test in January 2021, we transitioned from an in-person to a web-based approach due to COVID-19 pandemic control strategies.

After obtaining consent, we invited each eligible participant to LINE (LY Corporation) groups and divided them into small groups of 4-5 participants for convenience. The facilitated sessions were conducted in LINE group meetings with 4-5 participants each, led and guided by the certified resilience practitioner, who was also the first author (YCW). A third-party observer and assistant were present in each session. All participants received a weekly LINE message reminder to attend the sessions, regardless of their progress through the program. The purpose of these sessions was to debrief and help participants learn and demonstrate resilience skills, applying them to personal adversities in their daily lives. Additional details about the 4 sections are as follows:

- In the first section, we individually assessed 5 resilience protective factors and 2 resilience risk factors in the intervention group to understand the strengths and weaknesses of the participants. Asynchronous e-learning involved watching 2 resilience microfilms and completing a quiz with 5 questions related to the microfilms’ content using registered Google Forms (Alphabet Inc./Google LLC; see Multimedia Appendix 4) before the second section.
- In the second section, the situated practice was employed using a scenario involving an older adult who had just lost a significant other. Participants mutually role-played 2 roles: a low-resilience person struggling to cope with the loss, and a friend helping this person to clarify their needs and learn how to cope with a stressful life event. This included assessing available resources, locating support (family, social, and financial), and managing daily life. The tasks in this section included expressing negative feelings (the skill of coping), regaining control of one’s life (the skill of control belief), and participating in social activities to increase positive feelings in the future (the skill of manageability). During the session, participants were invited to share their relevant experiences, and the practitioner provided feedback and guidance on enhancing resilience skills based on each individual’s personal experiences.
- The third section, also utilizing situated practice, involved a scenario concerning an older adult who had just suffered paralysis after a stroke. Participants mutually role-played 2 roles: a low-resilience person unwilling to rehabilitate due to declining physical functioning and a friend providing help. The tasks in this section included accepting physical mobility impairments due to illness (the skill of coping), believing in oneself (the skill of control belief), and actively performing rehabilitation exercises (the skill of manageability). Similar to the second section, participants shared their personal experiences, and the practitioner provided individual feedback and guidance.
- In the final section, there was a review and practice of the 3 skills introduced in the 2 instructional scenarios, along with an evaluation of participants’ performance. Further recommendations were provided for participants who did not perform well. The practitioner used techniques such as reframing passive to active thoughts and guided participants to recognize their capabilities in adverse situations. Emphasis was placed on the importance and benefits of becoming a resilient older adult.

In the second and third sections, each participant alternately played the role of both the friend giving coping strategy advice and the low-resilience person, as assigned by the resilience practitioner. For example, a participant who played the friend giving advice in the scenario of spousal loss would then play the role of the low-resilience person in the disease-related event scenario, and vice versa.

### Evaluation Phase

To assess the efficacy of the intervention, outcome measures of participant engagement and feedback, including barriers to completion, were gathered through interviews and self-report feedback-marking sheets after the program. This focused specifically on evaluating the 3 resilience skills related to each scenario (see Multimedia Appendix 5).

### Control

To serve as a comparison to the web-based resilience-enhancing program, participants in the control group received usual care, which included patient education sheets compliant with National Health Insurance regulations (see Multimedia Appendix 6). They were instructed not to attend any resilience classes or programs.

### Outcome Measure

The key outcomes—resilience, physical activity, and well-being—were assessed at baseline (T0), 4 weeks (T1), and 12 weeks (T2) after completing the resilience program or after allocation. To facilitate participant convenience, telephone calls were made or stamped, self-addressed envelopes were provided for completing the self-assessed follow-up questionnaires. Additional telephone calls were made to participants who were unresponsive. Data were collected by a third party who was blinded to allocation. Demographic questionnaires, including information on age, gender, marital status, educational status, income, and other personal data, as well as details related to exercise habits such as type, frequency, and duration, were obtained for each participant.

Considering spousal loss as a stressful life event in our scenarios, it might be related to marital status among the participants. Therefore, we categorized marital status into 3 groups in our analysis: single and divorced, married, and widowed. This categorization accounts for potential differences in how individuals perceive and relate to the concept of spousal loss based on their marital status. For instance, individuals who are single or divorced may feel less connected to the concept compared with those who are married, where the scenario could be more stressful or tangible in practice. In addition, the impact of COVID-19 on monthly trends in older adults’ physical activity [39-42] was investigated and categorized into 3 groups: increase, relatively stable, and decrease (see Multimedia Appendix 7).

### Resilience

The Chinese version of the Resilience Scale (CRS) was chosen as the trial’s key outcome measure, considering Wagnild and Young’s [52] description of resilience as an internal resource akin to a protective factor. The Resilience Scale was originally developed to assess an individual’s abilities that enable them to cope and face adversity successfully [52], and it was translated into Chinese by Li [53]. The validated CRS consists of 25 items assessed on a 7-point Likert scale, serving as a self-report measure for individual resilience across 5 domains: self-reliance, perseverance, equanimity, meaningfulness, and existential aloneness (score range of 25-175). A higher score indicates better resilience. The CRS has been utilized in numerous studies involving the geriatric population [54], given that older adults commonly encounter challenging adversities.

### Physical Activity

Physical activity was assessed using the validated Chinese version of the Physical Activity Scale for the Elderly (PASE) [55]. Originally designed for large epidemiologic studies, the PASE measures the quantity and quality of physical activity performed by community-dwelling older adults in the previous week [56]. This assessment scale includes 12 activities that inquire about intensity, frequency, duration, and types of physical activity in recreational, household, and work-related settings, based on self-perceived performance. Each question’s score was assessed using a dichotomy and/or a 4-point Likert scale, and calculated by multiplying the time spent in recreational and work-related activities or participation in household activities by an empirically derived question weight (score range of 0-793). Higher scores indicate greater physical activity [57,58].

### Well-Being

Well-being was assessed using the validated Chinese version of the Well-Being Scale (WBS) [59], adapted from the Oxford Happiness Inventory developed by Argyle [60]. It comprises 24 questions rated on a 5-point Likert scale, evaluating 4 domains: life satisfaction, interpersonal relationships, self-assurance, and physical and moral integrity. The total score ranges from 24 to 120, with higher scores indicating greater well-being. The WBS has been widely utilized in geriatric research in Taiwan [61-63].

### Program Engagement and Feedback

Attendance of at least three sections was required for program completion. If a participant missed a session, they were invited to join another LINE group meeting for completion. Feedback data included interviews and self-report feedback-marking sheets. The interviews focused on participants’ experiences in completing the program, their level of engagement, preferred program elements, aspects they liked and disliked, and suggestions for program improvement. After program completion, interviews were conducted with all participants in the intervention group to analyze varying levels of program completion. Self-report feedback-marking sheets highlighted 3 key issues: participants’ confidence in coping (eg, facing and identifying problems, accepting current conditions), belief in themselves (eg, improving, receiving help, maintaining optimism), and ability to manage (eg, actively participating in physical and social activities, seeking support, knowing how to proceed) in adverse situations. A 4-point Likert scale was used, as indicated by Kusmaryono et al [64], to directly assess respondents on self-report feedback-marking sheets.

### Sample Size Calculation

G*Power version 3.1.9.2 (Department of Criminology, University of Melbourne) was used to calculate the sample size for an *F* test of analysis of variance with repeated measures, between factors. The required effect size was determined based on the recommendations of Bartholomaeus et al [65] and calculated using the Cohen equation [66], resulting in an effect size of 0.3. Assuming a power of 80%, an α of .05, and an effect size of 0.3, the initial estimated sample size required would be 52 participants. To account for anticipated loss to follow-up in the geriatric population, an additional 20% (10/52) was recruited, bringing the total sample size to a minimum of 62 participants.

### Statistical Analysis

We conducted intention-to-treat analyses on all participants (N=63), and missing data for all outcome measures were addressed through multiple imputations at the item level to minimize potential bias, following instructions reported previously [67,68]. IBM SPSS (version 24.0; IBM Corp.) was used to calculate demographics and outcome measures using means, SD, and frequencies. The significance of differences between groups at baseline was tested using independent samples *t*-tests and Pearson chi-square tests for ordinal and nominal variables, respectively. Group comparisons across the 3 time points were assessed using a 2-way repeated measures analysis of covariance. Additionally, the intraclass correlation coefficient was calculated to assess variance among time points, groups, and individuals. A linear mixed model was used to analyze the group-by-time interaction, with time points (T0, T1, and T2) and intervention (resilience vs control) as fixed effects, and individuals as a random effect, given that individual variability had the highest intraclass correlation coefficient ratio. Moreover, previous studies have demonstrated a significant impact of the COVID-19 pandemic on physical activity [31,40,42]. We accounted for the monthly trend of COVID-19 in Taiwan as a confounding factor in our analysis, categorizing it into 3 phases: escalation, stability, and decline of the epidemic. All analyses were adjusted for baseline values of the outcome measures, with statistical significance set at *P*≤.05.

Descriptive statistics were used to summarize the program engagement data. Qualitative thematic analysis of the interview data was conducted independently by 2 authors (YCW and SFS) with over 5 years of experience in gerontology. The analysis involved a systematic process: familiarization with the transcripts, in vivo coding, categorization, and development of overarching themes to closely examine and interpret the participants’ experiences with the resilience-enhancing program [69]. Three of the authors (YCW, SFS, and HHT) independently reviewed all final themes to ensure no significant patterns were overlooked.

### Ethical Considerations

The trial protocol was reviewed and approved by the Institutional Review Board of National Yang-Ming University (approval number YM108150E; February 4, 2020). The protocol was registered in the ClinicalTrials.gov database with the identifier NCT05808491. Informed consent was obtained individually offline before study participation, adhering to the principles of the Declaration of Helsinki. All data were deidentified to protect the privacy and confidentiality of participants. Participants received an NT $100 (US $3.08) coupon as compensation each time they completed the survey.

## Results

### Completion of Enrollment Procedures

**Figure 2:**
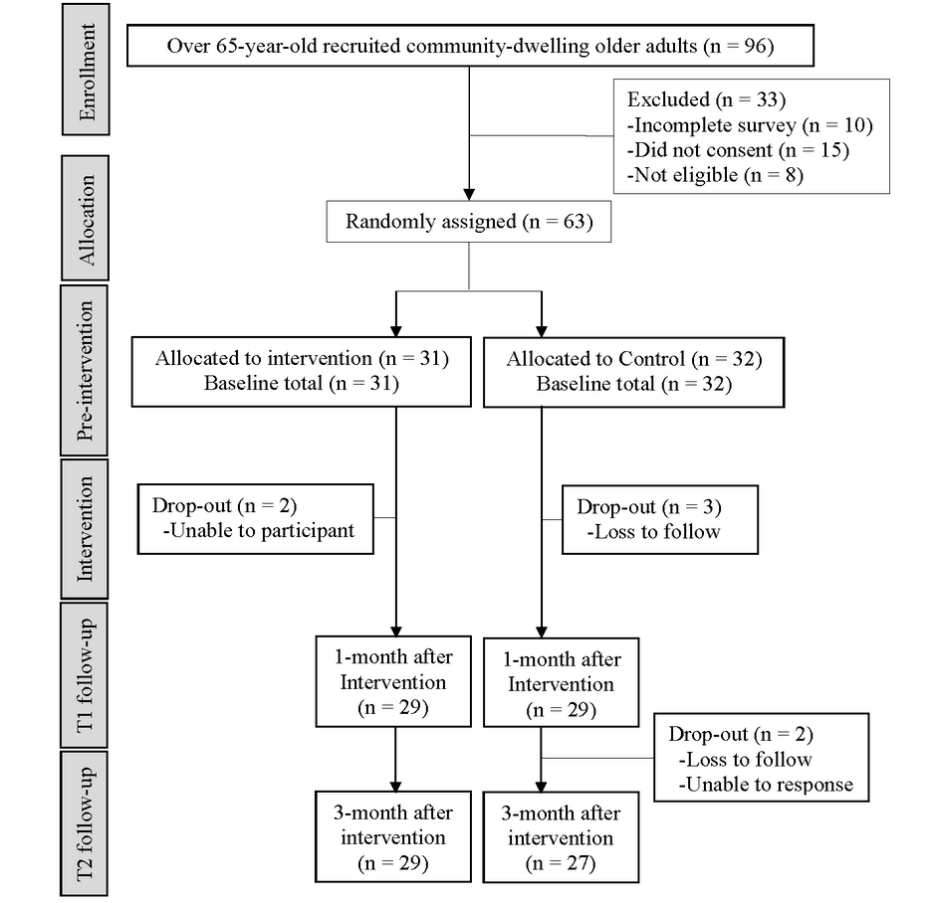
Consolidated Standards of Reporting Trial (CONSORT) diagram.

A total of 96 community-dwelling older adults underwent baseline assessment to determine eligibility. Of these, 63 eligible participants were randomly assigned to either the intervention group (n=31, 49%) or the control group (n=32, 51%) after completing the baseline assessment. All participants were contacted to complete follow-up measures at 4 and 12 weeks after the completion of the 4-week web-based resilience-enhancing program, regardless of their completion of previous measures. Figure 2 (also see Multimedia Appendix 8 [70]) presents the CONSORT (Consolidated Standards of Reporting Trials) diagram outlining the flow of participants throughout the study from February 2020 to December 2022.

### Missing Data

Of the 63 eligible participants included in the final analysis, 31 (49%) were in the intervention group and 32 (51%) were in the control group. In the intervention group, 94% (n=29) completed both the 4- and 12-week follow-up measures. In the control group, 84% (n=27) completed both follow-up measures, while 91% (n=29) completed only the 4-week follow-up measure. Three participants (9%) in the control group did not complete either the 4- or 12-week follow-up measures. The dropout rate was 6% (2/31) in the intervention group and 16% (5/32) in the control group.

### Baseline Characteristics

The baseline characteristics of the participants are presented in Table 1 for each group and for the entire sample. The mean age of participants was 72.11 (SD 5.65; range 65-92) years. Most participants were female (50/63, 79%), married (43/63, 68%), and had a habit of exercise (54/63, 86%). The majority engaged in walking (exercise), with a frequency of 3-5 times per week, and sessions lasting 30-60 minutes each. Characteristics were similar between groups; however, the mean age differed significantly between the intervention (69.27 years) and control (74.86 years) groups (t_45.6_=–4.53, *P*<.001). Table 2 presents bivariate correlations for all outcome variables at baseline for each group and the entire sample, indicating a positive association between resilience, physical activity, and well-being. Surprisingly, no relationship was found between resilience and physical activity within the control group.

**Table 1:** The demographic characteristics of participants.

Characteristics				Total (n=63)		Intervention (n=31)		Control (n=32)		*P*^a^ value
Number of participants randomized, n										
Age (years), mean (SD)				72.11 (5.65)		69.27 (3.08)		74.86 (6.23)		<.001
**Gender, n (%)**										.38
	Male		13 (21)		5 (16)		8 (25)			
	Female		50 (79)		26 (84)		24 (75)			
**Marriage status, n (%)**										.18
	Single and divorced		5 (8)		4 (13)		1 (3)			
	Married		43 (68)		21 (68)		22 (69)			
	Widowed		15 (24)		6 (19)		9 (28)			
**Educational status, n (%)**										.45
	Junior high school or below		22 (35)		9 (29)		13 (41)			
	High school		19 (30)		12 (39)		7 (22)			
	Associate’s degree and above		22 (35)		10 (32)		12 (38)			
**Family income per month, n (%)^b^**										.94
	<NTD 30,000		23 (37)		11 (35)		12 (38)			
	NTD 30,000-50,000		17 (27)		9 (29)		8 (25)			
	>NTD 50,000		23 (37)		11 (35)		12 (38)			
**Exercise habit, n (%)**										.18
	No		6 (10)		2 (6)		4 (13)			
	**Yes**									
		Walking	38 (60)		20 (65)		18 (56)			
		Gymnastics	7 (11)		1 (3)		6 (19)			
		Swimming	4 (6)		2 (6)		2 (6)			
		Other	8 (13)		6 (19)		2 (6)			
	**Frequency^c^**								.74	
		0-2 times/week	9 (14)		4 (13)		5 (16)			
		3-5 times/week	29 (46)		14 (45)		15 (47)			
		5-7 times/week	19 (30)		11 (35)		8 (25)			
	**Duration^c^**								.23	
		0-30 minutes/session	13 (21)		4 (13)		9 (28)			
		30-60 minutes/session	26 (41)		14 (45)		12 (38)			
		>60 minutes/session	18 (29)		11 (35)		7 (22)			

^a^*P* value between groups.

^b^NTD 1=US $0.031.

^c^A total of 57 participants had an exercise habit and were surveyed regarding the duration and frequency of their exercise habit.

**Table 2:** Correlation analysis of all outcome variables.

Outcomes				Total							Intervention							Control							
				Resilience		Physical activity		Well-being		Resilience			Physical activity		Well-being		Resilience			Physical activity		Well-being			
**Total**																									
	**Resilience**																								
		*r*	1		0.21^a^		–0.81^a^		—			—		—		—			—		—				
		*P* value	—^b^		<.001		<.001		—			—		—		—			—		—				
	**Physical activity**																								
		*r*	–0.21		1		–0.25^a^		—			—		—		—			—		—				
		*P* value	<.001		—		<.001		—			—		—		—			—		—				
	**Well-being**																								
		*r*	0.81		0.25		1		—			—		—		—			—		—				
		*P* value	<.001		<.001		—		—			—		—		—			—		—				
**Intervention**																									
	**Resilience**																								
		*r*	—		—		—		1			–0.34^a^		–0.64^a^		—			—		—				
		*P* value	—		—		—		—			<.001		<.001		—			—		—				
	**Physical activity**																								
		*r*	—		—		—		0.34			1		–0.42^a^		—			—		—				
		*P* value	—		—		—		<.001			—		<.001		—			—		—				
	**Well-being**																								
		*r*	—		—		—		0.64			0.42		1		—			—		—				
		*P* value	—		—		—		<.001			<.001		—		—			—		—				
**Control**																									
	**Resilience**																								
		*r*	—		—		—		—			—		—		1			–0.08		–0.90^a^				
		*P* value	—		—		—		—			—		—		—			.25		<.001				
	**Physical activity**																								
		*r*	—		—		—		—			—		—		0.08			1		—				
		*P* value	—		—		—		—			—		—		.25			—		—				
	**Well-being**																								
		*r*	—		—		—		—			—		—		0.90			0.14		1				
		*P* value	—		—		—		—			—		—		<.001			.05		—				

^a^The correlation is significant at a level of .05 (2-tailed).

^b^Not applicable.

In the intervention group, a majority reported positive outcomes in resilience factors: 74% (23/31) for family environment, 77% (24/31) for social integration, 81% (25/31) for positive and courageous coping strategies, 71% (22/31) for spiritual perspective, and 58% (18/31) for hope-derived meaning. Regarding risk factors, 55% (17/31) reported challenges with illness-related distress or defensive coping. Notably, more than one-third of participants reported difficulties in both illness-related distress and defensive coping, indicating potential negative impacts from chronic illness and defensive coping strategies.

### Outcomes and Estimation

#### Resilience

**Figure 3:**
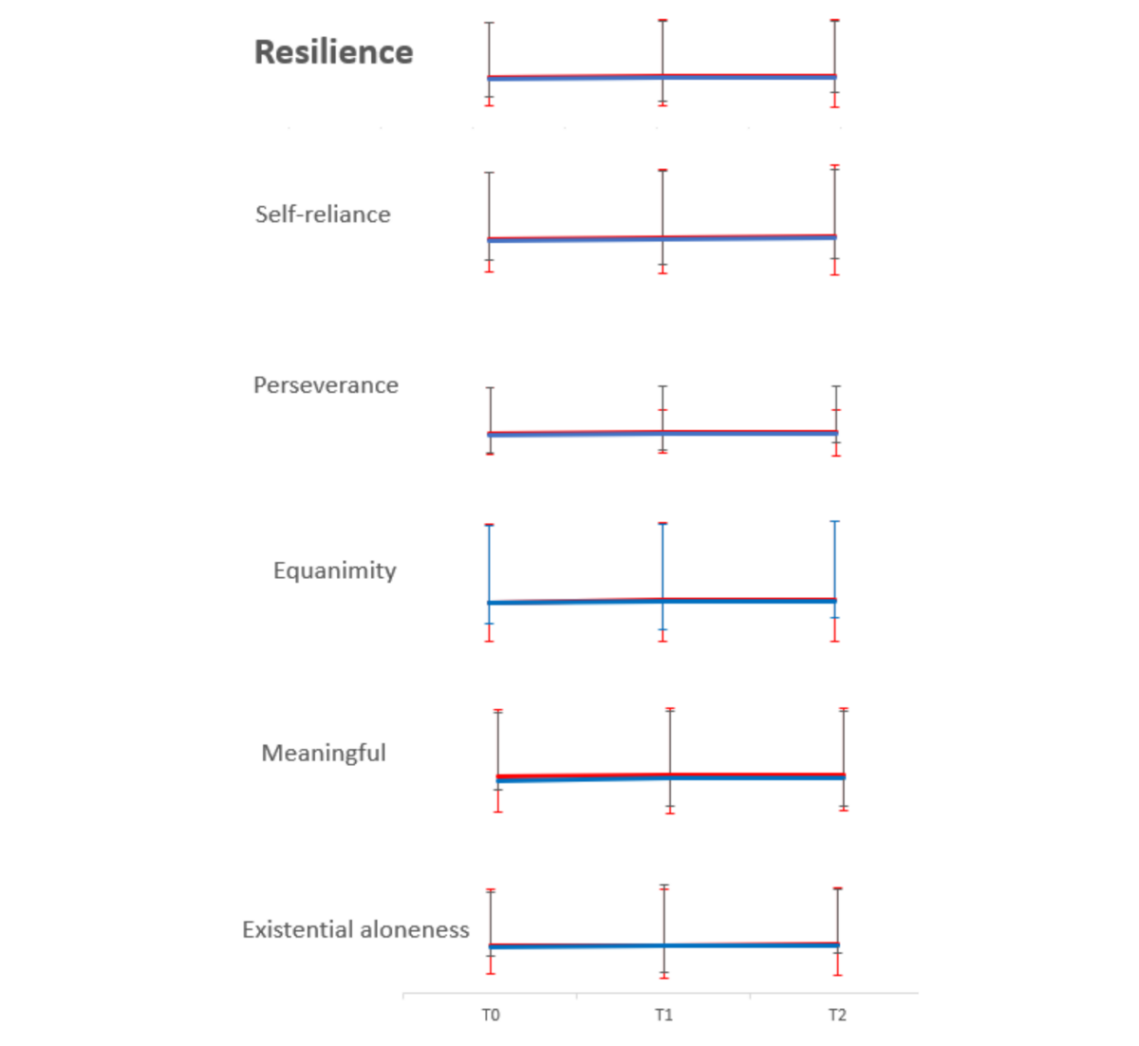
Estimated marginal mean score with error bars of resilience and its 5 domains over time. The red line represents the intervention group, while the blue line represents the control group. The mean scores of resilience in the intervention group were 138.84 (SD 22.51), 144.40 (SD 18.80), and 146.28 (SD 18.60), while in the control group they were 135.78 (SD 30.09), 139.29 (SD 27.61), and 140.62 (SD 29.05) at T0, T1, and T2, respectively. The mean scores of self-reliance in the intervention group were 33.29 (SD 6.28), 34.39 (SD 5.42), and 35.39 (SD 5.20), while in the control group they were 32.59 (SD 7.55), 33.69 (SD 6.80), and 34.70 (SD 6.98) at T0, T1, and T2, respectively. The mean scores of perseverance in the intervention group were 43.00 (SD 7.81), 46.01 (SD 6.72), and 46.00 (SD 7.01), while in the control group they were 42.91 (SD 9.29), 43.29 (SD 9.65), and 43.30 (SD 9.65) at T0, T1, and T2, respectively. The mean scores of equanimity in the intervention group were 33.19 (SD 5.80), 34.41 (SD 4.98), and 34.66 (SD 5.29), while in the control group they were 32.78 (SD 8.08), 33.36 (SD 7.81), and 33.42 (SD 7.92) at T0, T1, and T2, respectively. The mean scores of meaningful in the intervention group were 17.52 (SD 2.93), 18.00 (SD 2.32), and 18.01 (SD 2.63), while in the control group they were 16.22 (SD 4.48), 17.10 (SD 3.50), and 17.03 (SD 3.66) at T0, T1, and T2, respectively. The mean scores of existential aloneness in the intervention group were 11.84 (SD 2.40), 11.82 (SD 1.70), and 12.35 (SD 1.52) while in the control group they were 11.28 (SD 3.10), 11.94 (SD 2.27), and 11.85 (SD 2.53) at T0, T1, and T2, respectively.

These results reflect the intention-to-treat analysis, which included all participants regardless of missing data or intervention completion. The estimated marginal mean resilience scores over time are depicted in Figure 3 and Multimedia Appendix 9. At baseline, the resilience scores were 138.84 (SD 22.51) in the intervention group and 135.78 (SD 30.09) in the control group, ranging between 84 and 174 and 53 and 174, respectively. There was no significant between-group difference observed (t_61_=0.46, *P*=.65).

In the group-by-time interaction, there was no significant between-group difference observed over time in resilience (SE 7.49, 95% CI –10.74 to 18.61, *P*=.60), including across the 5 domains of self-reliance (SE 1.92, 95% CI –2.15 to 5.38, *P*=.40), perseverance (SE 2.62, 95% CI –4.86 to 5.40, *P*=.92), equanimity (SE 2.08, 95% CI –3.30 to 4.86, *P*=.71), meaningfulness (SE 0.92, 95% CI –1.53 to 2.08, *P*=.76), and existential aloneness (SE 0.63, 95% CI –1.22 to 1.24, *P*=.99), after controlling for age and marital status differences (Table 3).

**Table 3:** Mixed effects of the multilevel hierarchical model on resilience.

Outcome			Estimated marginal means		SE		95% CI		*P* value
**Resilience**									
	Intervention	3.94		7.49		–10.74 to 18.61		.60	
	Group × T1^a^	0.91		4.78		–8.46 to 10.28		.85	
	Group × T2^b^	2.77		6.04		–9.06 to 14.60		.65	
**Self-reliance**									
	Intervention	1.61		1.92		–2.15 to 5.38		.40	
	Group × T1^a^	0.07		1.23		–2.34 to 2.48		.96	
	Group × T2^b^	–0.18		1.49		–3.11 to 2.74		.90	
**Perseverance**									
	Intervention	0.27		2.62		–4.86 to 5.40		.92	
	Group × T1^a^	–0.07		1.86		–3.72 to 3.58		.97	
	Group × T2^b^	2.60		2.09		–1.50 to 6.70		.21	
**Equanimity**									
	Intervention	0.78		2.08		–3.30 to 4.86		.71	
	Group × T1^a^	0.14		1.68		–3.17 to 3.45		.93	
	Group × T2^b^	0.71		1.82		–2.85 to 4.28		.70	
**Meaningful**									
	Intervention	0.28		0.92		–1.53 to 2.08		.76	
	Group × T1^a^	0.15		0.62		–1.06 to 1.36		.80	
	Group × T2^b^	–0.29		0.87		–1.99 to 1.42		.74	
**Existential aloneness**									
	Intervention	0.01		0.63		–1.22 to 1.24		.99	
	Group × T1^a^	0.62		0.54		–0.44 to 1.68		.25	
	Group × T2^b^	–0.08		0.70		–1.45 to 1.30		.91	

^a^4 weeks after completing the resilience program

^b^12 weeks after completing the resilience program.

For group comparisons across the 3 time points, the results indicated no statistical significance in resilience (*F*_1,61_=0.49, *P*=.49), including across the 5 domains of self-reliance (*F*_1,61_=0.94, *P*=.34), perseverance (*F*_1,61_=0.29, *P*=.59), equanimity (*F*_1,61_=0.71, *P*=.40), meaningfulness (*F*_1,61_=0.03, *P*=.87), and existential aloneness (*F*_1,61_=0.07, *P*=.80).

#### Physical Activity

**Figure 4:**
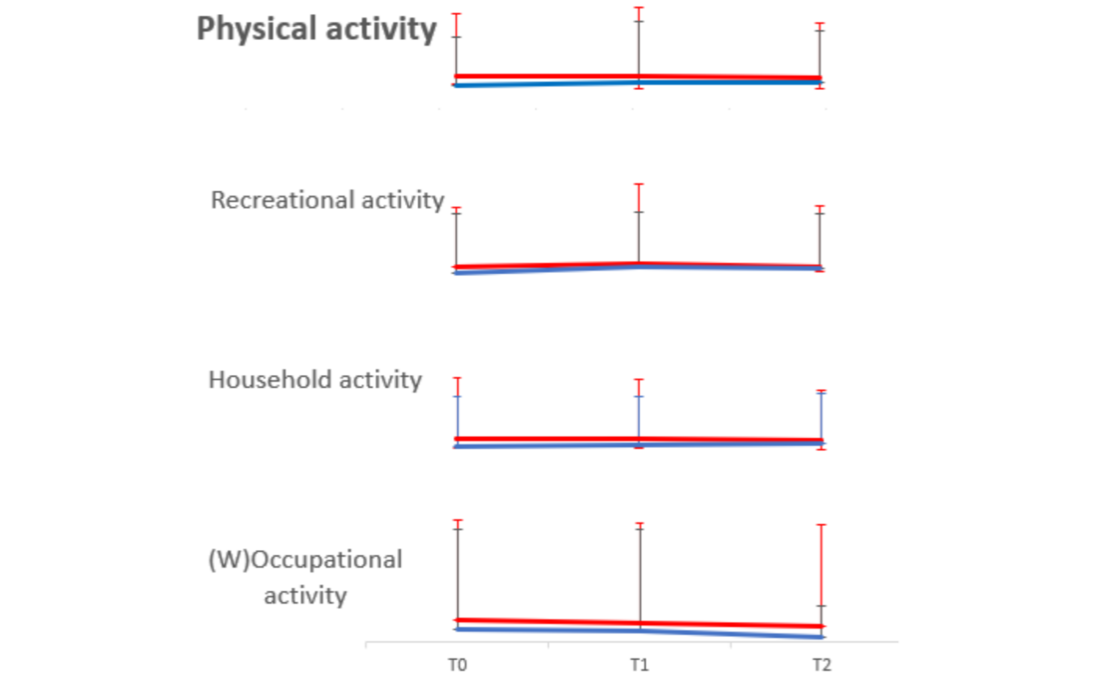
Estimated marginal mean score with error bars of physical activity and its 3 domains with weight occupational activity over time. The red line represents the intervention group, while the blue line represents the control group. The mean scores of physical activity in the intervention group were 162.16 (SD 76.25), 166.16 (SD 70.66), and 150.01 (SD 62.53), while in the control group they were 111.75 (SD 60.08), 127.68 (SD 88.59), and 122.75 (SD 68.85) at T0, T1, and T2, respectively. The mean scores of recreational activity in the intervention group were 50.06 (SD 44.10), 58.51 (SD 46.39), and 49.46 (SD 41.25), while in the control group they were 31.22 (SD 32.43), 46.63 (SD 50.85), and 41.99 (SD 41.28) at T0, T1, and T2, respectively. The mean scores of household activity in the intervention group were 86.25 (SD 35.63), 85.14 (SD 24.17), and 80.66 (SD 33.09), while in the control group they were 66.00 (SD 38.73), 66.98 (SD 36.87), and 74.19 (SD 37.31) at T0, T1, and T2, respectively. The mean scores of weighted occupational activity in the intervention group were 25.84 (SD 30.76), 22.52 (SD 24.17), and 19.89 (SD 24.61), while in the control group they were 14.55 (SD 29.66), 14.07 (SD 24.71), and 6.57 (SD 10.71) at T0, T1, and T2, respectively.

The estimated marginal mean score of physical activity over time is shown in Figure 4 and Multimedia Appendix 9. At baseline, the scores in the intervention and control groups were 162.16 (SD 76.25) and 111.75 (SD 60.08), respectively, ranging between 46 and 369 and 0 and 278, respectively. There was a significant between-group difference (t_61_=2.92, *P*=.005).

In the group × time interaction, there was no significant between-group difference over time in the change of physical activity (SE 15.18, 95% CI –24.74 to 34.74, *P*=.74), including 3 domains: recreational activity (SE 8.58, 95% CI –15.77 to 17.87, *P*=.90), household activity (SE 8.23, 95% CI –4.92 to 27.35, *P*=.17), and occupational activity (SE 5.56, 95% CI –18.13 to 3.66, *P*=.19). This analysis controlled for age difference, marital status, exercise habits, and COVID-19 monthly trends (Table 4 and Multimedia Appendix 7).

**Table 4:** Mixed effects of multilevel hierarchical model on the change of physical activity.

Outcome		Estimated marginal means	SE	95% CI	*P* value
**Physical activity**					
	Intervention	5.01	15.18	–24.74 to 34.76	.74
	Group × T0^a^ to T1^b^ duration	8.73	20.06	–30.59 to 40.05	.66
**Recreational activity**					
	Intervention	1.05	8.58	–15.77 to 17.87	.90
	Group × T0^a^ to T1^b^ duration	7.81	13.84	–19.31 to 34.93	.57
**Household activity**					
	Intervention	11.21	8.23	–4.92 to 27.35	.17
	Group × T0^a^ to T1^b^ duration	–9.93	10.91	–31.33 to 11.46	.36
**Occupational activity**					
	Intervention	–7.23	5.56	–18.13 to 3.66	.19
	Group × T0^a^ to T1^b^ duration	10.01	7.23	–4.17 to 24.18	.17

^a^Baseline.

^b^4 weeks after completing the resilience program.

For group comparisons at 3 time points, the results showed no statistical significance in physical activity (*F*_1,61_=1.71, *P*=.20), including 3 domains: recreational activity (*F*_1,61_=0.40, *P*=.53), household activity (*F*_1,61_=2.14, *P*=.15), and occupational activity (*F*_1,61_=0.30, *P*=.59), after controlling for baseline differences.

#### Well-Being

**Figure 5:**
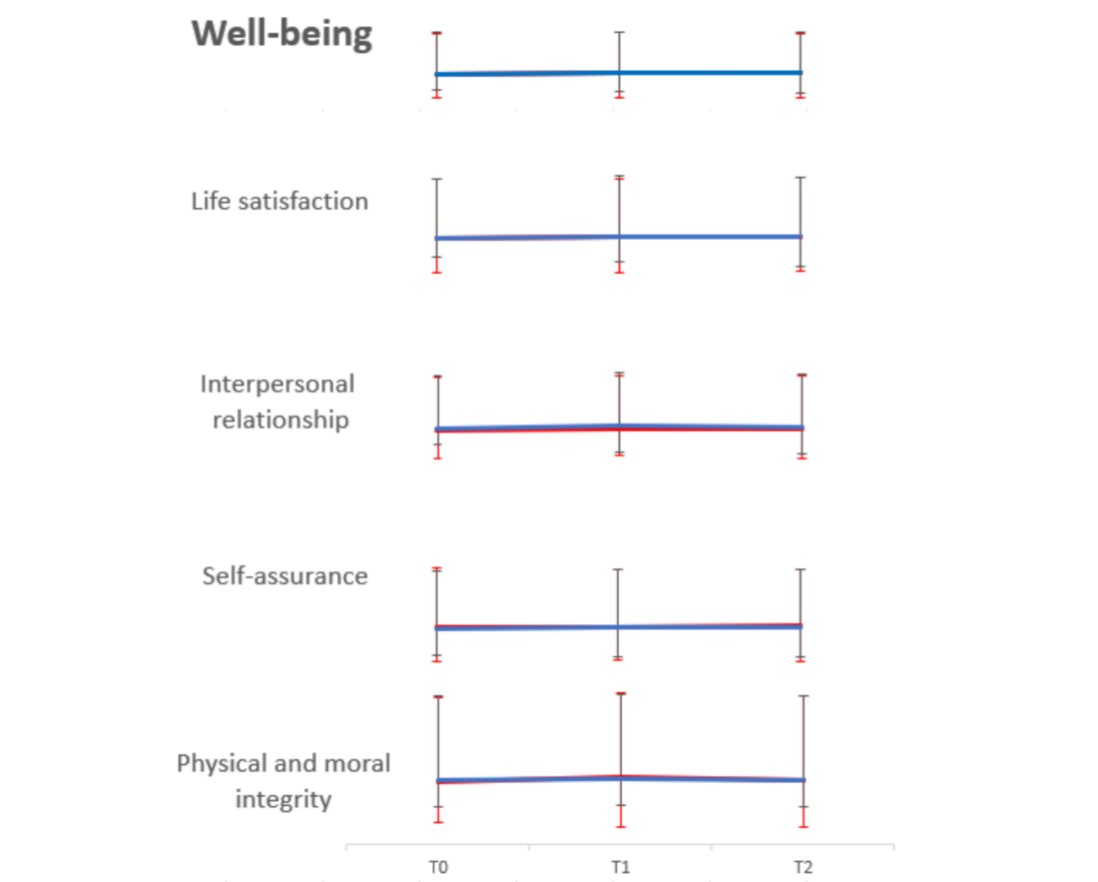
Estimated marginal mean score with error bars of well-being and its 4 domains over time. The red line represents the intervention group, while the blue line represents the control group. The mean scores of well-being in the intervention group were 92.29 (SD 12.69), 95.65 (SD 11.09), and 94.23 (SD 10.73), while in the control group they were 92.28 (SD 17.15), 96.29 (SD 17.38), and 95.22 (SD 15.63) at T0, T1, and T2, respectively. The mean scores of life satisfaction in the intervention group were 27.13 (SD 3.90), 27.84 (SD 3.61), and 28.06 (SD 3.82), while in the control group they were 27.06 (SD 5.54), 28.54 (SD 5.32), and 28.36 (SD 4.51) at T0, T1, and T2, respectively. The mean scores of interpersonal relationship in the intervention group were 22.74 (SD 3.54), 23.83 (SD 3.19), and 23.88 (SD 3.35), while in the control group they were 23.38 (SD 5.13), 24.88 (SD 4.81), and 24.22 (SD 4.16) at T0, T1, and T2, respectively. The mean scores of self-assurance in the intervention group were 23.94 (SD 3.23), 23.62 (SD 2.55), and 24.14 (SD 2.87), while in the control group they were 22.88 (SD 4.11), 23.38 (SD 4.15), and 23.53 (SD 4.57) at T0, T1, and T2, respectively. The mean scores of physical and moral integrity in the intervention group were 18.48 (SD 3.19), 19.82 (SD 2.78), and 18.81 (SD 2.87), while in the control group they were 18.97 (SD 4.48), 19.60 (SD 4.69), and 19.07 (SD 4.27) at T0, T1, and T2, respectively.

The estimated marginal mean score of well-being over time is shown in Figure 5 and Multimedia Appendix 9. At baseline, the scores in the intervention and control groups were 92.29 (SD 12.69) and 92.28 (SD 17.15), respectively, ranging between 71 and 119 and 46 and 120, respectively. There was no statistically significant difference between the groups (t_61_=0.002, *P*=.99).

In the group × time interaction, there was no significant between-group difference over time in well-being (SE 3.74, 95% CI –2.68 to 11.98, *P*=.21), including the 4 domains of life satisfaction (SE 1.23, 95% CI –1.11 to 3.70, *P*=.29), interpersonal relationships (SE 1.06, 95% CI –0.84 to 3.30, *P*=.24), self-assurance (SE 0.97, 95% CI –0.85 to 2.94, *P*=.28), and physical and moral integrity (SE 1.07, 95% CI –0.95 to 3.25, *P*=.28), after controlling for age difference and marital status (Table 5).

**Table 5:** Mixed effects of multilevel hierarchical model on well-being.

Outcome		Estimated marginal means	SE	95% CI	*P* value
**Well-being**					
	Intervention	4.65	3.74	–2.68 to 11.98	.21
	Group × T1^a^	–0.30	2.07	–4.36 to 3.77	.89
	Group × T2^b^	–0.81	2.89	–6.49 to 4.87	.78
**Life satisfaction**					
	Intervention	1.30	1.23	–1.11 to 3.70	.29
	Group × T1^a^	0.20	0.85	–1.48 to 1.87	.82
	Group × T2^b^	–0.33	1.06	–2.41 to 1.74	.75
**Interpersonal relationship**					
	Intervention	1.23	1.06	–0.84 to 3.30	.24
	Group × T1^a^	0.78	0.64	–0.48 to 2.05	.22
	Group × T2^b^	0.34	0.88	–1.38 to 2.01	.70
**Self-assurance**					
	Intervention	1.05	0.97	–0.85 to 2.94	.28
	Group × T1^a^	–0.57	0.68	–1.92 to 0.77	.40
	Group × T2^b^	–1.03	0.83	–2.65 to 0.60	.22
**Physical and moral integrity**					
	Intervention	1.14	1.07	–0.95 to 3.25	.28
	Group × T1^a^	–0.70	0.75	–2.19 to 0.78	.35
	Group × T2^b^	0.21	0.77	–1.30 to 1.71	.79

^a^4 weeks after completing the resilience program

^b^12 weeks after completing the resilience program.

For group comparisons at 3 time points, the results showed no statistical significance in well-being (*F*_1,61_=1.84, *P*=.18), including the 4 domains of life satisfaction (*F*_1,61_=1.79, *P*=.19), interpersonal relationships (*F*_1,61_=2.88, *P*=.10), self-assurance (T0: *F*_1,61_=0.24, *P*=.63; T1: *F*_1,61_=0.28, *P*=.60; T2: *F*_1,61_=3.43, *P*=.07), and physical and moral integrity (*F*_1,61_=1.28, *P*=.26).

### Program Feedback

Program feedback is summarized in Table 6. Out of the 31 participants randomized to the intervention group, 29 (94%) provided feedback on the program. Among them, 24 (83%) completed the entire program. For those who did not complete it, 2 of 29 (7%) cited illness or impaired function as the main barrier. Encouragingly, all participants who provided feedback reported having the most confidence to use the skills in control belief outside of the program. Additionally, 26 out of 29 (90%) expressed confidence in using coping skills, while 24 out of 29 (83%) felt confident in applying manageability skills.

**Table 6:** Program completion, barriers to engagement, and feedback.

Program feedback			Participants
**Percentage of program completed, n (%)**			
	100%		24 (83)
	75%		5 (17)
**Reasons for noncompletion, n (%)**			
	Lack of time to complete the program		1 (3)
	Illness impaired function to complete the program		2 (7)
	Competing demands, such as work		1 (3)
	Forgot to complete the program		1 (3)
**Have confidence in using program strategies outside the program, n (%)**			
	**In coping**		
		Most confidence and above	26 (90)
		Some confidence and below	3 (10)
	**In control belief**		
		Most confidence and above	29 (100)
		Some confidence and below	0 (0)
	**In** **manageability**		
		Most confidence and above	24 (83)
		Some confidence and below	5 (17)

During the interviews, older participants expressed appreciation for the opportunity to participate in this facilitated program. They found the mentorships invaluable for practicing resilience skills in the face of adversity, particularly in overcoming challenges posed by the COVID-19 pandemic. Participants highlighted their learning from personal and others’ experiences. The program implementation was noted as the most significant benefit for participants.

This course was very good and I learnt a lot from either role-play or experience shared by others. Now I learnt the ways from others’ experience and the practitioner taught to help one of my relative get out of the lost after her husband died. She committed suicide after lost her husband. I didn’t know how to help her at that time, but, I do now.

Patient #9, female

Moreover, the flexibility of the web-based digital intervention format allowed participants to attend sessions without direct contact and interact online. YouTube and LINE emerged as the most popular and familiar social media platforms. Older participants found it relatively easy to watch microfilms and participate in group meetings for program completion, even amid quarantine or symptoms of chronic disease.

Furthermore, several participants referenced prior experiences of feeling powerless when trying to support a grieving friend who had lost a spouse. They expressed frustration when their efforts to provide companionship were met with negative emotions directed inward by their friend. Additionally, those with depressive tendencies recounted instances of using defensive coping mechanisms that led them to withdraw from family and friends. One older participant shared the sentiment that “time heals all wounds,” while another mentioned considering becoming a monk for spiritual support after their spouse passed away.

When my husband who has been in a vegetative state for many years passed away, I lost my focus in my life but felt relieved at the same time. It took me a year and half to overcome, and I had been thought about to becoming a monk. My children and friends supported me a lot during that period. I truly didn’t want to bother them too much at that moment, but I appreciated their company to heal me. Now I know how to help myself recover sooner, and know the ways to help others based on my own experience and the skills learning from this program.

Patient #2, female

Another benefit of this facilitated program related to disease-related events is practicing mindset transformation. To assist individuals experiencing depressive states, we practiced shifting focus from physical aspects to resetting goals aimed at quickly regaining health by identifying what is most important:

Think about your family and children, which is the most important things for you, work hard to restore your health, change your mind toward a positive attitude, and find specific solutions to regain health. Most people discouraged in rehab period. You can start with a goal you want to achieve in the next weeks to build your confidence.

Patient #5, male

In addition, the program raised awareness among participants about the importance of vigilant self-care.

This program was considered ideal as it provided community-dwelling older adults with opportunities to practice and develop resilience skills, preparing them to face adversity effectively. Enhancing resilience is crucial for preventing and mitigating the severity of mental and physical health issues, especially among the geriatric population:

This course reminded me the ways to build resilience and practiced the skills constantly. It now was in my mind. When encountering similar problems in the future, I can use them without hesitation.

Patient #26, female

Several older participants suggested improvements such as delivering the program on devices with larger screens that are familiar to older adults, such as tablets or computers. They also recommended providing clear instructions on how to join the correct group and participate in sessions with shared screens, individually guiding them step by step before each session starts. Older participants who initially struggled with operating the device were assessed by the practitioner, observer, and their family. One participant who suggested computer-based delivery felt it would improve accessibility to the program content, especially if delivered on a mobile device:

My eyesight isn’t good. It hard for me to read the sentences in the scenario via mobile device, so if I was able to assess it through a big screen that would be easier for me.

Patient #14, female

## Discussion

### Principal Findings

This study investigated the efficacy of a web-based resilience-enhancing program aimed at improving resilience, physical activity, and well-being in community-dwelling older adults. Participants in the resilience-enhancing program were compared with a control group, with postintervention assessments conducted at 4 and 12 weeks after program completion or allocation. No statistically significant between-group differences were observed over time in resilience, physical activity, and well-being after controlling for baseline differences. Interestingly, despite a small number of participants withdrawing from the study initially, all intervention group participants completed the program and both follow-up assessments, indicating high study participation and adherence rates. Participant feedback underscored the significance of enhancing resilience to cope with challenges in later life. All intervention group participants valued the resilience-enhancing program, noting its benefits in improving resilience and well-being. The findings also highlighted both the opportunities and challenges of internet-based programs for the geriatric population. Participant feedback emphasized the necessity for targeted interventions tailored to promote healthy aging.

### Comparison With Prior Work

Our findings underscore the acceptability and feasibility of implementing a web-based resilience-enhancing program for community-dwelling older adults, a need identified in previous literature [7-13]. This trial was initially conducted in North Taiwan at the onset of the COVID-19 pandemic, necessitating a shift from in-person to web-based strategies for COVID-19 control. Subsequently, we conducted the study during the peak of the pandemic, when substantial damage to the physical, mental, emotional, and social dimensions of older adults was evident [31,39-42]. The geriatric population showed significant demands for recovering their health afterward [8], highlighting the potential need and responsibility for public service interventions. Despite these challenges, we successfully recruited participants, implemented the web-based resilience-enhancing program as planned, and collected data at the designated time points. Therefore, our study has provided initial evidence that implementing web-based resilience-enhancing programs with similar design features for the geriatric population is feasible. These findings pave the way for further research to design appropriate web-based resilience programs aimed at improving health and well-being in older adults.

The web-based resilience-enhancing program has demonstrated acceptability among the geriatric population in community settings, as evidenced by the low dropout rate among participants. In our study, the dropout rate in the intervention group was 6% (2/31), consistent with rates reported in prior studies utilizing virtual interventions [71], underscoring its high level of acceptance [72]. This was further confirmed by program feedback from participants in the intervention group, demonstrating high engagement and satisfaction among community-dwelling older adults. These findings underscore the genuine demand for thoughtfully evaluated web-based resilience-enhancing programs for implementation across community settings. Additionally, the dropout rate in the control group was 16% (5/32), which was more than double that of the intervention group. However, the dropout rate in our study was relatively low compared with other RCTs [73]. The disparity in dropout rates between the intervention and control groups may be attributed to the older age and poorer health status of participants in the control group [73]. Reasons for dropout included “loss to follow-up” and “inability to respond,” as indicated in the CONSORT diagram, during the COVID-19 pandemic.

Encouragingly, feedback from participants in the intervention group indicated satisfaction with the 2 distinct components of the resilience-enhancing program. The web-based facilitated sessions and mentorship were particularly well received. For example, practitioners offered individual guidance on coping strategies, belief control, and managing personal bereavement. This was especially meaningful for widowed participants who shared their experiences of spousal loss, while married participants were invited to discuss related concerns during the sessions. The effectiveness of mentorship in enhancing resilience has been supported by prior research focused on resilience interventions for older adults [74,75]. These findings underscore mentorship support as a pivotal element in bolstering resilience among the geriatric population. Future studies could explore the specific impacts of individual versus group mentorship sessions, considering their potential flexibility and adaptation to the diverse physical conditions of older adults. Interviews conducted with community-dwelling older adults in the participating trust highlighted a demand for resilience training tailored to this population. Participants identified the necessity for supportive measures to mitigate various stressors and promote healthy aging [39]. These insights suggest that the design features, content, and format of the resilience-enhancing program are suitable and well-received within this demographic, indicating potential for replication and adaptation in other populations. Furthermore, the findings of this study regarding design, engagement, and acceptability represent a significant extension of prior work. These results are promising, especially for low-cost programs that are relatively accessible to this target group.

There is limited research examining the impact of similar interventions, making direct comparisons of long-term effects in the geriatric population challenging. Despite hypothesizing that resilience, physical activity, and well-being would differ between groups after an intervention, our study did not find evidence to verify these mechanisms through the RIM. The program’s ineffectiveness in increasing resilience could potentially be attributed to the necessity for participants to encounter triggering events, such as new episodes of functional deficits, the death of a spouse, retirement, or financial exploitation, to demonstrate resilience despite acquiring coping strategies during the intervention. However, we did not collect data on such triggering events. Another potential reason could be related to certain risk factors affecting older adults’ learning, such as insufficient time to practice learned material, cognitive declines, and limitations in information-processing capabilities [76]. For future research aiming to quantify confidence and satisfaction after program completion in the intervention group, it is suggested to implement strategies such as pre- and postsurveys to assess the application of learned strategies.

In addition, previous research has shown that the COVID-19 pandemic led to a general decrease in physical activity during lockdown [37]. Our study was conducted during the height of the pandemic, and it revealed varying levels of physical activity among participants, particularly in the control group. This underscores the need to consider the potential impact of the pandemic as a contributing factor to the observed lack of intervention effect on physical activity. Furthermore, we observed a lack of relationship between resilience and physical activity within the control group, which contrasts with findings from prior studies [32,33]. This discrepancy may be attributed to the diverse physical activity levels among our control group participants, who engaged in both active and sedentary lifestyles and utilized different aspects of resilience, as noted by Wermelinger et al [33]. Moreover, the substitution of the in-person approach with a web-based format may have affected the intervention’s effectiveness on physical activity outcomes. Future research could benefit from closely tracking changes in physical activity in response to web-based resilience-enhancing practices.

### Strengths and Limitations of the Study

To our knowledge, this study represents the first implementation of a web-based, role-play, and talk-in-interaction resilience-enhancing program for community-dwelling older adults during the COVID-19 pandemic, underscoring the benefits of such interventions for promoting healthy aging [10-12]. Furthermore, the high participation and adherence rates, along with positive qualitative feedback received, highlight the successful engagement of participants and provide valuable insights into essential elements for designing and implementing digital programs tailored to community-dwelling older adults. This study lays an important foundation and offers clear guidance for optimizing web-based programs and research designs in the future, particularly in terms of ensuring the suitability of equipment for the geriatric population.

One limitation of our study was the control group design, which received standard care without social components such as virtual meetings or educational sessions with the same frequency as the intervention group. This discrepancy may have contributed to the double dropout rate observed in the control group compared with the intervention group. In addition, the study was conducted only in North Taiwan, where participants who were willing to join may have had higher social involvement [75]. The study also had a relatively small sample size and an uneven distribution of age and physical activity at baseline between groups, potentially impacting the generalizability of the findings. Attrition bias was introduced by a higher attrition rate than previously reported for older adults [12]. Future studies should aim to include participants from a broader geographical range and diverse backgrounds to enhance the generalizability of the findings.

Moreover, the outcome measures in our study were self-assessed, which could be subject to participant bias. We did not assess objective measures of health status, such as body mass index, fasting lipid profile, blood pressure, heart rate, serum creatinine, presence of other comorbid conditions, number of prescribed medications, and surgery history. Therefore, we were unable to comment on whether these variables may have influenced the intervention outcomes. Finally, conducting a study during the COVID-19 pandemic may have influenced the processes and findings of this study. Initial recruitment progress was not as anticipated, and vulnerable older adults were disproportionately affected in health, social, and economic dimensions during this global public health crisis [8,30]. Moreover, the intervention was shifted to a web-based method as part of COVID-19 control strategies, which may have influenced participant responses. These factors should be considered when implementing similar resources across health and social care settings in the postpandemic era.

### Conclusions

This RCT provides compelling evidence regarding the study design, engagement, and acceptability of a web-based resilience-enhancing program for community-dwelling older adults. Furthermore, the study underscores the significance and necessity of tailored resilience-enhancing programs for older adults living independently, who encounter challenges in late adulthood and would benefit from accessible forms of structured support for healthy aging. Additionally, participants highlighted the importance of mentorship components in the web-based resilience-enhancing program, which played a pivotal role in its successful delivery. This suggests that mentorship could be an area of future research focus and practical application. Further studies should build on these findings by optimizing program designs to ascertain whether such interventions can lead to meaningful improvements in pursuing healthy aging among this target population.

## Appendix

Multimedia Appendix 1 Resilience practitioner training.

Multimedia Appendix 2 Preintervention survey—resilience factors.

Multimedia Appendix 3 Links to the 2 microfilms on YouTube.

Multimedia Appendix 4 Quiz on the 2 microfilms.

Multimedia Appendix 5 Postintervention survey.

Multimedia Appendix 6 National Health Insurance regulation documents of education sheets.

Multimedia Appendix 7 2020 Taiwan COVID-19 monthly trend.

Multimedia Appendix 8 CONSORT-eHEALTH (V 1.6.1) checklist.

Multimedia Appendix 9 Results of the estimated marginal mean score of resilience, physical activity, and well-being over time.

## Abbreviations

CONSORT: Consolidated Standards of Reporting Trials
CRS: Chinese version of the Resilience Scale
PASE: Physical Activity Scale for the Elderly
RCT: randomized controlled trial
RIM: Resilience in Illness Model
WBS: Well-Being Scale
WHO: World Health Organization

## Data Availability

The data that support the findings of this study are available from the corresponding author upon reasonable request.
