# Supplementary material for: A Web-Based Resilience-Enhancing Program to Improve Resilience, Physical Activity, and Well-being in Geriatric Population: Randomized Controlled Trial
Source: J Med Internet Res. 2024 Jul 25;26:e53450. doi: 10.2196/53450 (PMC11310648; doi:10.2196/53450)
Supplement: Multimedia Appendix 2 [file jmir_v26i1e53450_app2.pdf]

Multimedia Appendix 2

<u>Preintervention Survey-</u> resilience factors

Item	3 Excellent	2 Good	1 Fair	0 Poor
Protective factor				
1. Family Environment<br>Family members communicate with each other smoothly and can conduct a consistent consensus.				
2. Social Integration<br>Having friends you can talk to, taking advice from others and participating in group or community activities.				
3. Positive and Courageous Coping<br>Be able to maintain a positive attitude towards emergencies, and face it bravely, resolve it actively.				
4. Spiritual Perspective<br>Be yourself and believe in yourself				
5. Hope-derived Meaning<br>Always get your hope up and feel the future to be bright				
Risk factor				
6. Illness-related Distress<br>Easy to have negative emotions when unexpected events occur, such as feeling lost, sad or anxious.				
7. Defensive Coping<br>Worry about others' opinions and easy to fear of and deny new things.				
