# Supplementary material for: A Web-Based Resilience-Enhancing Program to Improve Resilience, Physical Activity, and Well-being in Geriatric Population: Randomized Controlled Trial
Source: J Med Internet Res. 2024 Jul 25;26:e53450. doi: 10.2196/53450 (PMC11310648; doi:10.2196/53450)
Supplement: Multimedia Appendix 3 [file jmir_v26i1e53450_app3.docx]

## <https://www.youtube.com/watch?v=beQzMM_FEy0&t=13s>, <https://www.youtube.com/watch?v=yfcmXnkuFuo&t=27s>
