# Supplementary material for: A Web-Based Resilience-Enhancing Program to Improve Resilience, Physical Activity, and Well-being in Geriatric Population: Randomized Controlled Trial
Source: J Med Internet Res. 2024 Jul 25;26:e53450. doi: 10.2196/53450 (PMC11310648; doi:10.2196/53450)
Supplement: Multimedia Appendix 4 [file jmir_v26i1e53450_app4.pdf]

Multimedia Appendix 4

Quiz of the two microfilms

1. Can resilience help you effectively resolve crises and turn them into opportunities?
   ( ) Yes
   ( ) No
2. Does highly resilient person act like a deflated ball?
   ( ) Yes
   ( ) No
3. Which is a way to increase resilience?
   ( ) (1) Social contact
   ( ) (2) Crisis management
   ( ) (3) Positive thinking
   ( ) (4) All of the above
4. You are a highly resilient person. What would you do first if your spouse passed away?
   ( ) (1) Achieve goals
   ( ) (2) Social contact
   ( ) (3) The most important thing is
   ( ) (4) Growth mindset
5. When you feel uncomfortable, do you face it with positive attitude or ignore it?
   ( ) (1) Face it with positive thoughts
   ( ) (2) Ignore it
