# Supplementary material for: A Web-Based Resilience-Enhancing Program to Improve Resilience, Physical Activity, and Well-being in Geriatric Population: Randomized Controlled Trial
Source: J Med Internet Res. 2024 Jul 25;26:e53450. doi: 10.2196/53450 (PMC11310648; doi:10.2196/53450)
Supplement: Multimedia Appendix 5 [file jmir_v26i1e53450_app5.pdf]

Multimedia Appendix 5

Postintervention Survey- feedback sheet

Please read each question carefully and rate it by circling a number based on how confident you are that you can complete the behaviour if you lost a spouse.

Item	3 Totally confident	2 Most confident	1 Some confident	0 Absolutely no confident
**Coping**				
1. I can accept that emergencies happen.				
2. I can discuss my emotions with family and friends.				
3. I don't run away from problems.				
**Control belief**				
1. I believe I can get better.				
2. I am willing to accept help from others.				
3. I can stay optimistic.				
**Manageability**				
1. I can actively participate in group activities every day.				
2. I am willing to seek out people or groups that I can support actively.				
3. I know how to do it.				

Please read each question carefully and rate it by circling a number based on how confident you are that you can complete the behaviour if you have impaired physical mobility.

Item	3<br>Totally confident	2<br>Most confident	1<br>Some confident	0<br>Absolutely no confident
**Coping**				
1. I can figure out what my current problem is.				
2. I can handle my negative emotions				
3. I can accept impaired physical mobility caused by illness				
**Control belief**				
1. I believe I can get better				
2. I am willing to accept help from others				
3. I can stay optimistic				
**Manageability**				
1. I can actively perform rehabilitation exercises				
2. I am willing to participate actively				
3. I know how to do it				
