# Supplementary material for: A Web-Based Resilience-Enhancing Program to Improve Resilience, Physical Activity, and Well-being in Geriatric Population: Randomized Controlled Trial
Source: J Med Internet Res. 2024 Jul 25;26:e53450. doi: 10.2196/53450 (PMC11310648; doi:10.2196/53450)

健康老化

銀髮族保健手冊

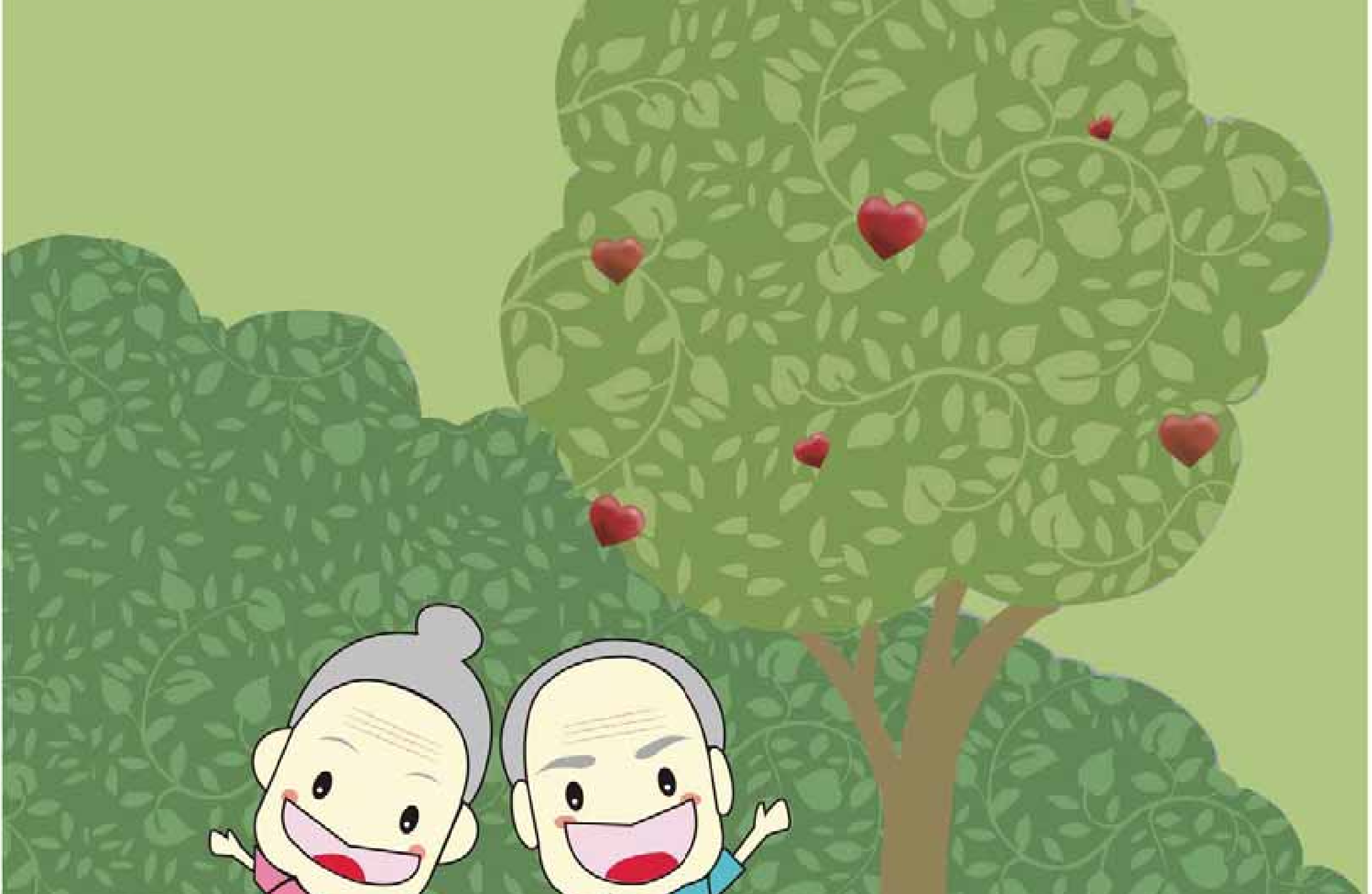

行政院衛生署國民健康局 關心您

健康老化

銀髮族保健手冊

著者：行政院衛生署國民健康局

出版機關：行政院衛生署國民健康局

出版年月：99 年 12 月

主題 頁碼

主題		頁碼
再版序		1
理事長序		2
一、健康老化		3
二、健康生活型態	均衡飲食	5
	理想體重	7
	不吸菸、不喝酒、不嚼檳榔	9
	運動休閒生活	12
	如何抒解壓力	15
三、自我健康管理	健康檢查	17
	視力保健	19
	口腔保健	20
	服藥須知	22
	預防接種小叮嚀	24
四、常見慢性病的自我管理	高血壓	26
	高血脂	28
	糖尿病	31
	腦中風	33
	退化性關節炎	35
五、老年症候群	骨質疏鬆症	37
	跌倒	39
	長期失眠	41
	打鼾	43
	尿失禁	44
	失智症	46
六、安居宜養	老人住宅	48
	社會參與	50
	機構式照護	51
七、附錄：老人照護資訊		53
編輯資訊		55

再版序

台灣老年人口比率逐年增加，至民國99年7月，老年人口已達247萬6千人，占總人口10.7%，約每10人就有1位老人；又從先進國家人口老化速度來看，我國老化速度名列前茅，與日本相同。隨著人口的老化，相繼而來的通常是慢性疾病及失能人口的增加，醫療保健照護資源大量的被使用等，所以如何增進長者自我健康照顧的知能，預防疾病或延緩病程，讓長者活的健康、動的安全，享受健康快樂的老年生活，是我們努力的目標。

爰此，本局前委託台灣老年醫學會研發本手冊，手冊內容淺顯易懂，適合一般民眾參閱。然因應健康照護資訊的進步及考量實務現況，本次主要增修食物採買與保存、運動安全小叮嚀、活動要訣、血糖正常值及治療目標值、尿失禁防治、高血脂的藥物治療、酒精與藥物的交互作用..等內容，讓本手冊更貼近了老年人的生活需求。

感謝所有參與本教材修正之專家、學者，在編修工作上的用心，提供寶貴修正意見及圖片，使本手冊得以順利付梓。同時期盼您能與親朋好友、左鄰右舍一同分享，一同為健康而努力，讓大家都能活得久久，健康久久。

行政院衛生署國民健康局　局長

邱 淑 媞 謹識

中華民國99年12月

理事長序

隨著台灣人口急速老化，國人如何因應老化社會的來臨是半刻不容緩的事情。提升國內老年醫療品質以及建構完善老年照護體系一直是台灣老年醫學會努力的目標，因此，提供民眾正確的健康觀念、提醒民眾提早做好老年生活準備，台灣老年醫學會責無旁貸。

老化不可避免，如何讓老年生活更健康、更有尊嚴，營造彩色的老年生活才是最重要的。本手冊的完成是結合了老年醫學各領域的專家，包括醫師、護理、營養、衛教等，為的就是要提供即將進入老年以及已經進入老年的民眾和家屬，自我健康管理以及醫療與保健上的專業建言。

在此感謝行政院衛生署國民健康局給予本學會研發本手冊的機會，感謝所有協助完成銀髮族保健手冊的專家學者。大家共同為中老年的健康促進所做的努力，得以將本手冊呈現給親愛的您。請不吝給予指教。

台灣老年醫學會理事長

陳 慶 餘

健康老化

隨著醫藥衛生的進步，國內老年人口日益增加，民國九十八年國人平均餘命已達男性78.9歲、女性82.5歲，可以說已經邁入高齡化的社會。長壽不是唯一的健康老化指標，世界衛生組織針對人口老化的趨勢，提出活躍老化的基本原則，希望退休的老人與失能老人，皆能夠繼續參與家庭、同儕及社區的活動，使老化成為正面的經驗。

生老病死是人生必經的階段，在老化過程中，預防疾病、延遲失能和規劃身後事準備，都必須融入老年生活來身體力行，在策略上需建構對於老人的支持性環境，整合資源，增進老人的飲食、運動、社會關係與就業等機會，並提供老人整合性的健康照護，以改善老年人的整體生活機能。

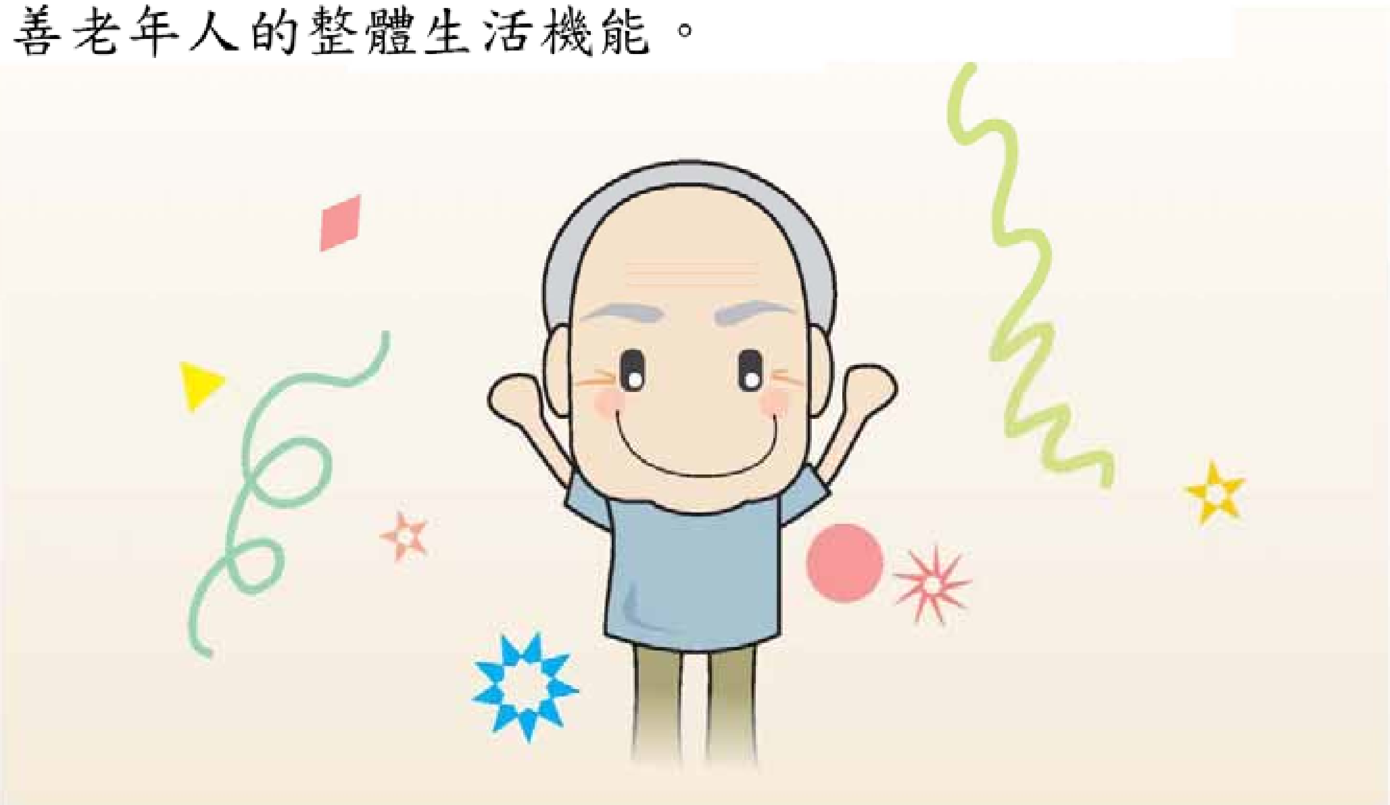

健康老化的條件有三

1. 減少疾病危險因子和失能

如不吸菸、控制體重、接種疫苗、預防跌倒等

2. 強化身體和心理能力

如經常運動、均衡飲食、感恩惜福、培養興趣等

3. 積極從事社會生活

如志工服務、與人為善、敦親睦鄰、多做公益等

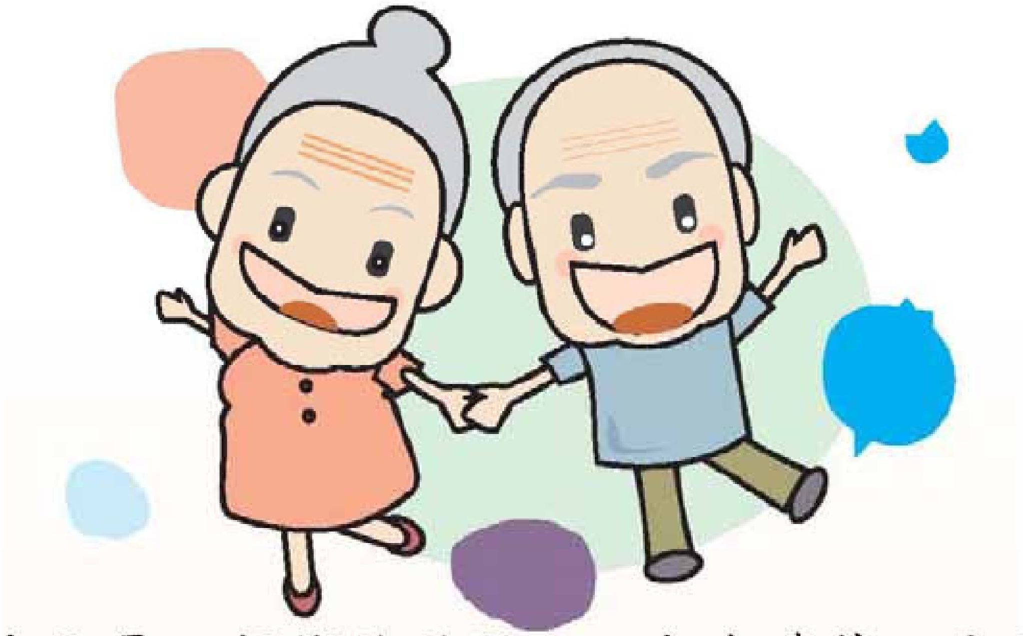

老化是一個複雜的過程，包含遺傳、生活型態、慢性疾病等許多因素交錯作用，都將影響老化過程的健康狀況。研究發現，維持規律的身體活動，有良好的認知能力和保持適當的營養狀況，是延緩衰老的不二法門，也是預防銀髮族發生跌倒、住院、併發症、失能和死亡的重要關鍵。我們衷心期望在寶島台灣的每一位銀髮族，在活躍老化的進程中，皆能充滿活力，過著有創造力和有智慧的健康生活。

均衡飲食

常見的進食問題?

銀髮族會因漸漸老化，咀嚼、吞嚥與對食物的味覺下降；再加上消化能力減弱，備餐與採購的意願及體力較不如前，而易導致進食不佳與營養不良。此外，由於代謝力下降，如果加上少動偏食，也容易造成體重過重，而導致心血管和代謝功能異常，則罹患慢性病的風險增加，對健康和生命有嚴重的威脅。

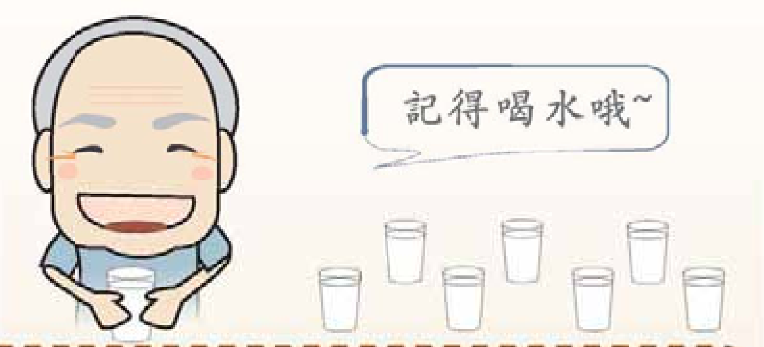

您每天要如何做?

- 營養均衡、多蔬果、多喝水、少油、少糖、少鹽
- 多吃不同顏色組合的蔬果。
- 全麥或是糙米要占穀物攝取的一半量。
- 少吃高熱量低營養或是油脂含量高的食物(可樂、奶茶與零食)。
- 注意隨時補充水分，因為年齡增加會降低口渴的感覺。
- 增加纖維的攝取可以降低便秘，例如吃水果儘量不去皮、全麥飲食、吃水果取代果汁。

每日飲食建議量

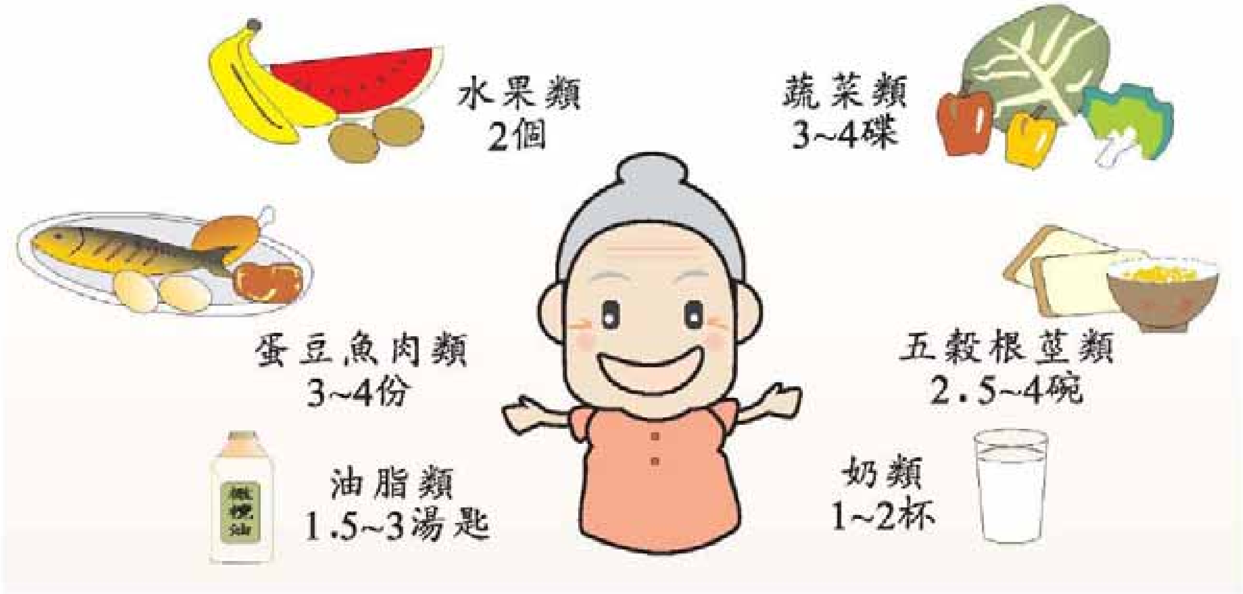

食物採買與保存

隨著年齡增加，嗅覺、味覺與眼力都下降，對於食物的新鮮度判斷能力也下降。因此要注意生熟食的刀具要分開、避免生食、冰箱內的食物都貼上保存日期、吃不完的食物最好在兩個小時內放入冰箱。

等量代換參考

蛋豆魚肉類	每份=蛋1個=豆腐1塊=魚肉1兩=瘦肉1兩
五穀根莖類	1碗飯=2碗稀飯=2碗麵條=1個中型饅頭
蔬菜類	1碟=100公克=煮熟後的量為半碗
水果類	1個=約柳丁大小
油脂類	1湯匙=15公克
奶類或水份	1杯=240cc

理想體重

維持理想體重是維護身體健康的基礎

體重與健康有密切關係，體重過重容易引起糖尿病、高血壓和心血管疾病等慢性病；體重過輕會使抵抗力降低，容易感染疾病，故平日應養成均衡的飲食習慣，及持續有恆的運動，以確保健康。

您的理想體重是多少?

理想體重的算法：

理想體重(公斤)＝22×身高(公尺)^2

理想體重範圍為理想體重±10%

例：男性身高170公分

理想體重(公斤)＝22×1.7^2＝63.6公斤

成年人之理想體重範圍：

身高（公分）	理想體重範圍（公斤）	身高（公分）	理想體重範圍（公斤）
145	39.0	150	41.5-54.0
155	44.5	160	47.0-61.5
165	50.0	170	53.5-69.0
175	56.5	180	60.0-77.5

避免腹部肥胖

腰圍過粗的人，比較容易得到「糖尿病」、「高血壓」、「高血脂症」、「心臟病及腦中風」的機率，分別是一般健康民眾的6、4、3、2倍。

腰圍過粗：以正確的方法測量出的腰圍值為

男性≧90公分(35吋半)

女性≧80公分(31吋半)

成人腰圍測量方法

1. 除去腰部覆蓋衣物，輕鬆站立，雙手自然下垂。
2. 以皮尺繞過腰部，調整高度使能通過左右兩側腸骨上緣至肋骨下緣之中間點(如圖)，同時注意皮尺與地面保持水平，並緊貼而不擠壓皮膚。
3. 維持正常呼吸，於吐氣結束時，量取腰圍。

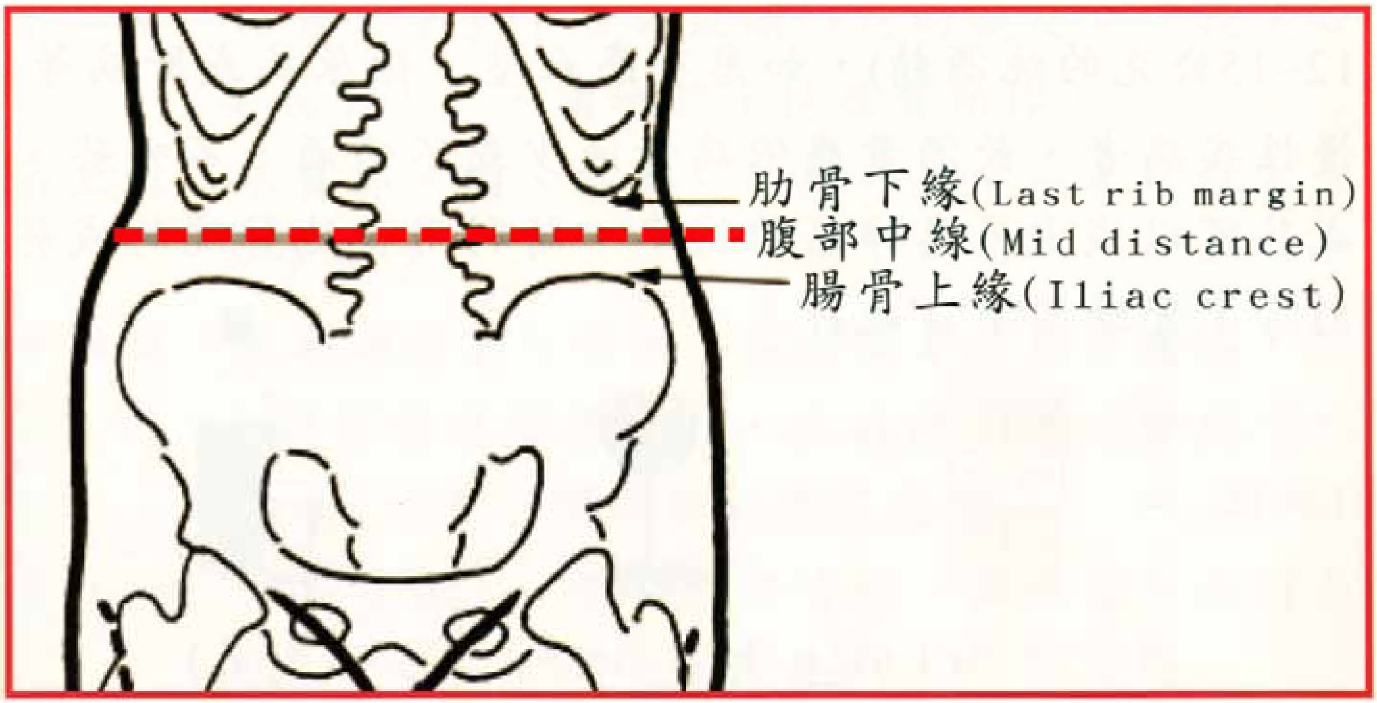

不吸菸、不喝酒
不嚼檳榔

吸菸、過量飲酒、嚼檳榔對健康有什麼影響?

吸菸易導致肺癌、口腔癌或胃癌等多種癌症，並減低肺功能，造成慢性阻塞性肺病(COPD)，及傷害心臟血管等。

飲酒過量容易導致肝硬化、心臟及心血管疾病、癌症或胰臟炎等，酒精易與多種藥物發生交互作用，增加發生不良反應的危險，這些問題在銀髮族較年輕人更為嚴重。

嚼檳榔會造成牙齒磨損及牙周病，同時也會使口腔內膜組織纖維化，導致罹患口腔癌的危險增加，有嚼食檳榔、吸菸且飲酒的人與完全都沒有的人比較，得到口腔癌的風險高出123倍!

隨著年齡的增加，身體的水分含量也隨著下降，因此，老人比較容易累積血中的酒精濃度。而由於代謝率下降，排泄功能老化，老年身體解酒需要較長的時間，也增加酒醉的危險性。

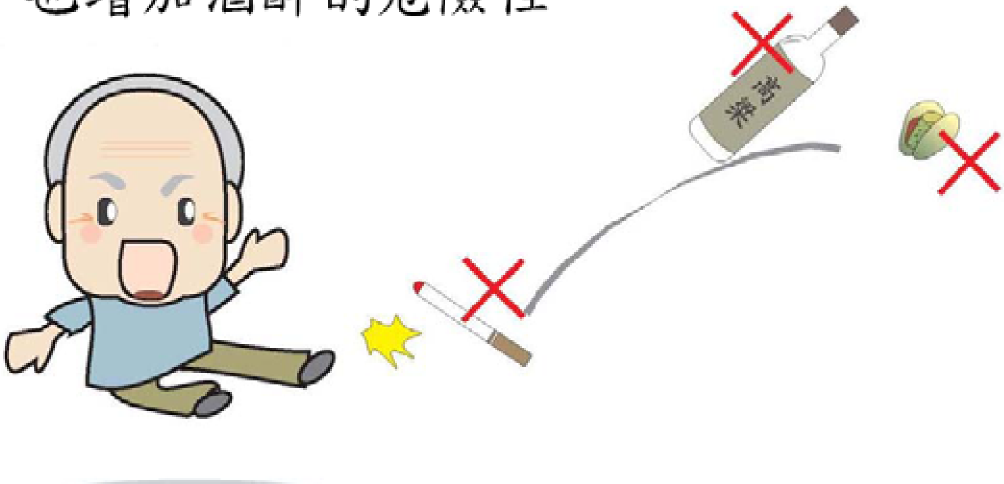

常見的酒精與藥物交互作用包含：

1. 阿斯匹靈類的藥物或是關節炎的止痛藥會和酒精交互作用，容易造成腸胃出血。
2. 流鼻水感冒藥物，會讓人昏昏欲睡，加上酒精會造成嗜睡與協調力下降。
3. 安眠藥、鎮靜劑與酒類混用，會減少老人的防禦力，增加老人意外傷害可能性。
4. 酒精與高血壓、糖尿病、痛風、心臟病的藥物併用，會降低藥效，加重原有症狀。

最後請記住，老人代謝藥物與酒精的速度較慢，所以就算是間隔了數個小時，上述的酒精與藥物交互作用仍會發生。

如何預防菸、酒、檳榔對健康的傷害?

對銀髮族來說，平均每天飲酒量不宜超過1單位（約12～15公克的純酒精），如患有高血壓、糖尿病或肝病等慢性疾病者，飲酒量應依病情減少或不喝酒、不抽菸。戒菸可以減少罹患肺癌的機率，請利用當地醫院的戒菸門診，幫助您早日戒菸。

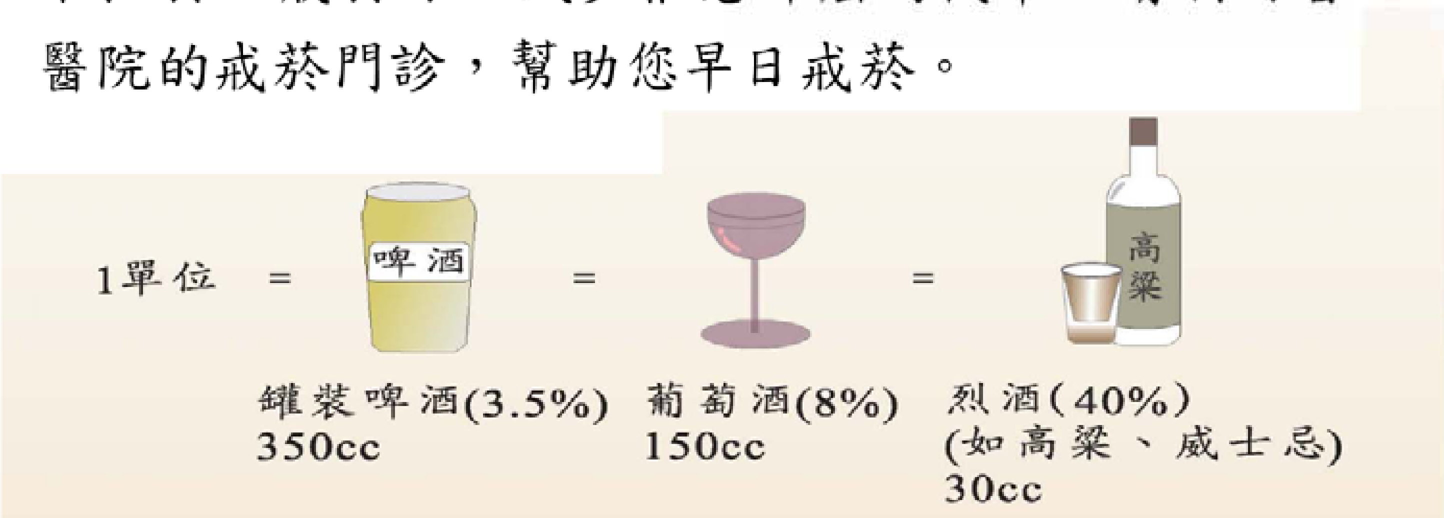

口腔內如出現紅色或白色斑塊、久治不癒的潰瘍、容易出血、臉頰出現腫塊、口腔附近麻木、疼痛或張口困難，可能是口腔癌的徵兆，請找耳鼻喉科醫師或牙醫師幫忙診斷及治療。

拒絕菸、酒、檳榔的六招

第一招「挑戰自己」：下定決心，訂一個日子如生日開始戒除。

第二招「昭告天下」：告訴最關心您的親友：「我決心要戒了！」，請他們支持及幫助你。

第三招「不買菸、酒、檳榔」：想一想，一年可以省下多少錢?

第四招「避開誘惑」：口袋、家裡及車內皆不備菸，外出時盡量選擇禁菸區，在戒掉期間減少與有吸菸、飲酒和有嚼檳榔的親友接觸。

第五招「豬羊變色」：用無糖口香糖、白開水，或以唱歌、深呼吸代替。

第六招「尋找救兵」：請教您的家庭醫師。

運動休閒生活

運動有規律，元氣一把罩

不管年紀多大，運動及身體活動都是對你有益處的，可以持續幫助您：

- 維持並提升身體機能，讓您可以保持獨立。
- 讓您有更多的活力去做您想做的事。
- 改善您的平衡。
- 預防或是延遲疾病發生，像是心臟病、糖尿病、乳癌及大腸癌及骨質疏鬆。
- 振奮精神和避免憂鬱。

事前有準備，安全沒煩惱

活動前填寫『體能活動前簡易自我評量表』。有任一項答「是」，應禁止激烈活動並與醫師討論後方可進行。

評量題目	是
是否有醫師告訴您，心臟有問題，只能做醫師建議的運動?	
活動時是否會有胸痛的感覺?	
過去幾個月來，是否曾在未活動的情況下出現胸痛的情形?	
是否曾有因暈眩而失去平衡或意識的情況?	
是否有骨骼或關節問題，可能因活動而惡化?	
是否有因高血壓或心臟疾病而需要服藥治療(醫師處方)?	
是否知道有任何不適合活動的原因?	

運動安全小叮嚀

- 從"慢"做起，特別是沒有活動習慣的人，在一開始從事身體活動時從小地方做起，一點一滴累積您的活動量及活動難度。
- 不要憋氣，您可以在用力時"吐氣"，放鬆時"吸氣"。
- 使用安全設備，例如穿著安全的鞋子。
- 除非你的醫師告訴你要限制液體攝取外，一般人在活動時應攝取足夠量液體。
- 背部挺直，收肚子(收下腹)。
- 做伸展動作前肌肉先暖身。

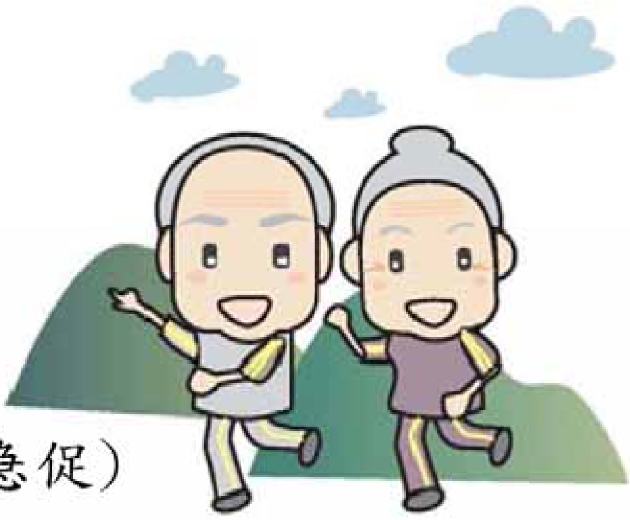

活動四要訣

- 喘(增加心肺耐力，呼吸有點急促)
- 力(運用肌肉，增加肌肉強度)
- 平(左右腳交替、訓練平衡)例如：左右交替單腳站立、不用手從椅子上站起、腳尖抵腳跟走路)
- 伸(伸展動作可以增加您的身體彈性)
- 依據下圖『體能活動建議金字塔』以加強第一、二類並適度增加第三、四類運動為主。

你準備好了嗎？就從今天開始吧！

左右交替單腳站立

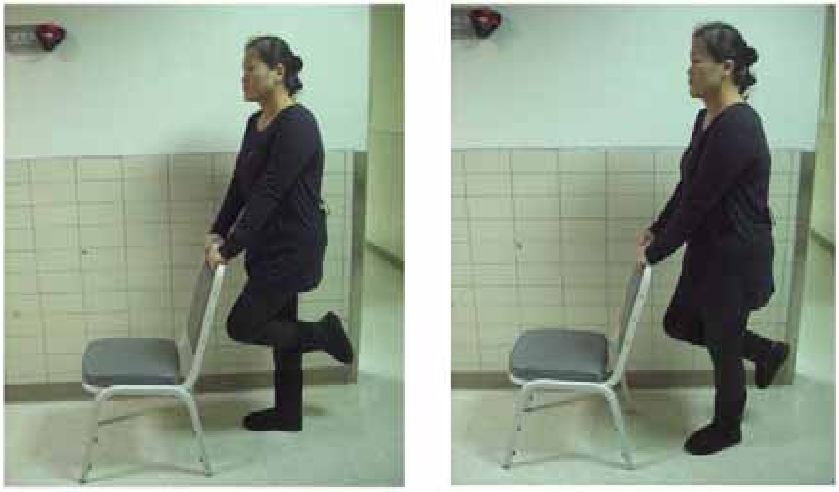

不用手從椅子上站起

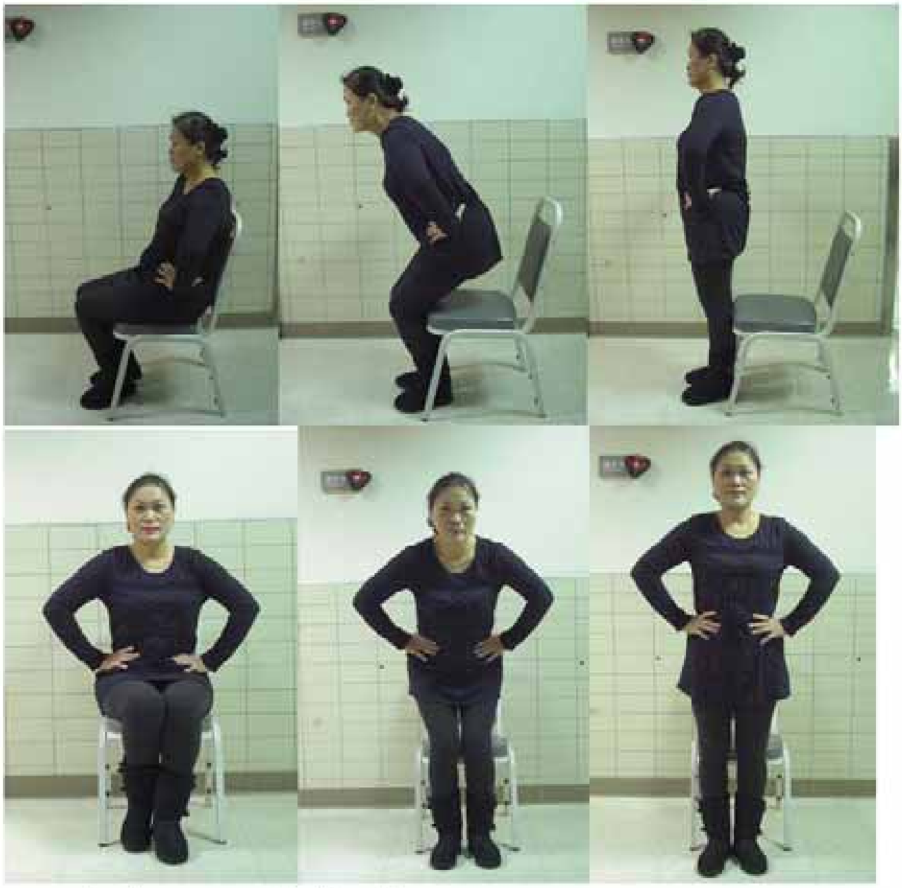

腳尖抵腳跟走路

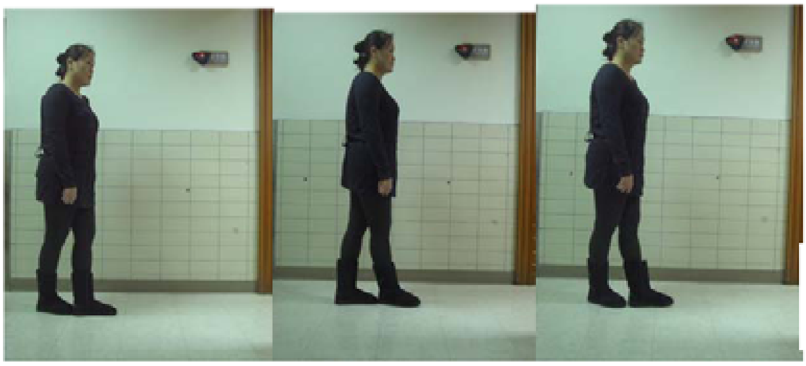

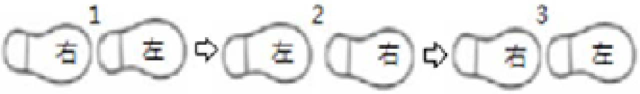

如何抒解壓力

老年的壓力來源?

壓力是現代人常見的問題。人活在慣性裏，當需要改變既有的習慣，壓力就隨之而生。常見壓力的生理反應包括：心跳加速、血壓升高、頭頸僵硬、呼吸加快、口乾等。

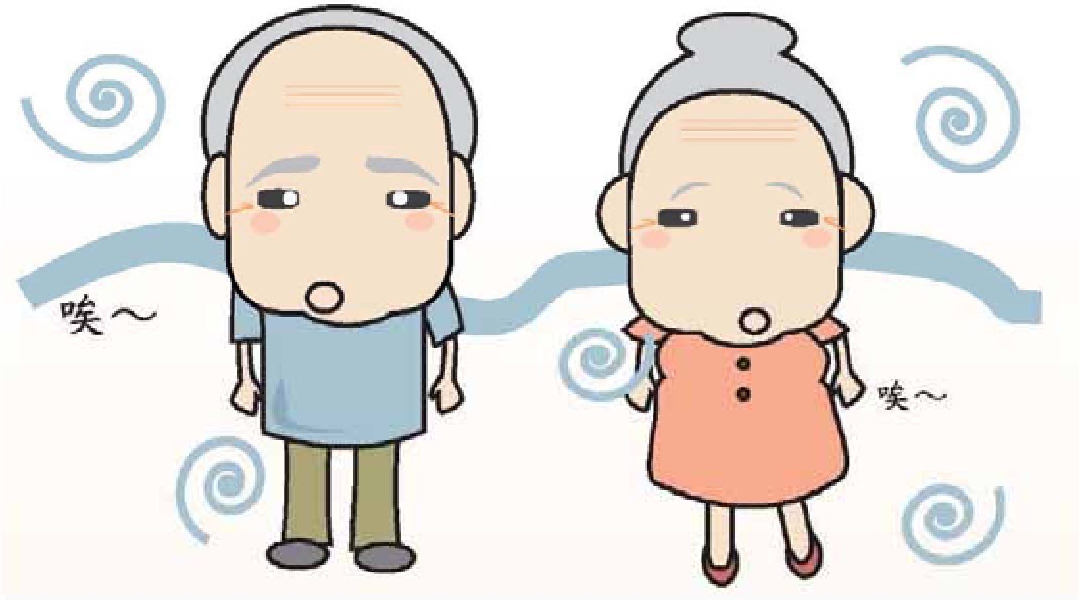

老年人常會因為下列的因素而造成壓力：

1. 慢性多重疾病
2. 身體功能受限
3. 慢性疼痛
4. 退休
5. 經濟壓力
6. 照顧壓力
7. 遷居
8. 喪偶或是哀傷
9. 認知功能改變，包含記憶力下降
10. 嚴重的疾病(癌症、失智)

壓力大引起的身體不舒服有那些?

- 緊張(焦慮)、憂鬱、情緒不穩定、容易生氣
- 頭痛、頸部僵硬、肩膀酸痛、腰酸背痛、胸悶胸痛、全身酸痛
- 食量過多或減少、腸胃不適、便秘或腹瀉
- 心悸、畏寒、身體發熱、易流汗及頻尿
- 失眠或睡得比平常多、容易疲勞

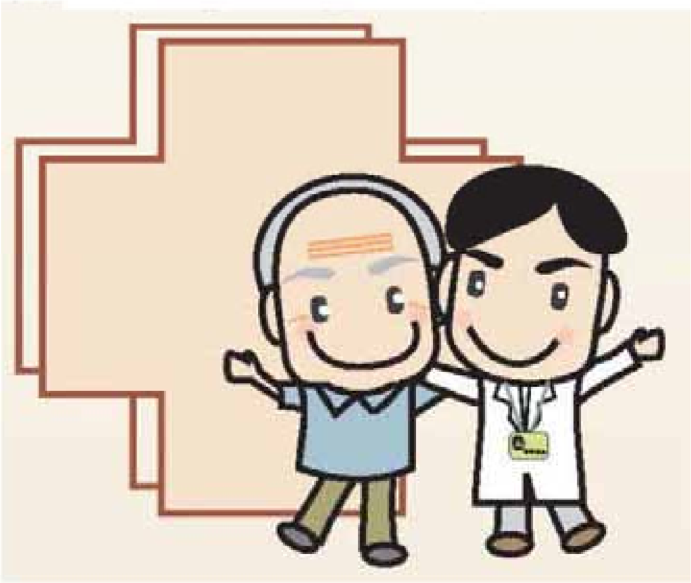

如何抒解壓力?

老人壓力調適的秘訣包含

1. 多參與社區或團體活動
2. 培養健康規律的生活習慣
3. 參與喜愛的活動
4. 正向的思考
5. 借鏡過去成功的調適方式
6. 學習放鬆的技巧(深呼吸、肢體伸展等)
7. 照顧者壓力可以透過參加支持成長團體來得到協助。
8. 如果上述方式無法抒解，請尋求專業協助。

健康檢查

健康檢查重要嗎?

有些銀髮族對健康檢查存有戒心，認為「不知影，好了了」或「沒檢查沒病，一檢查便到處都是病」。其實，我們的身體像汽車一樣，開了多年後，可能多少有些損壞或老化，故需定期到廠檢查保養，身體也是「有保養，不一樣喔」。

健康檢查可以查出所有疾病嗎?

沒有任何的檢查可診斷出所有的疾病，健康檢查目的是要把已有變化的疾病或癌症，提早在沒有不舒服時就先找出來，這叫做「早期診斷」；已有不舒服時去做健康檢查是不適當的，這時候應找醫師看診才對。

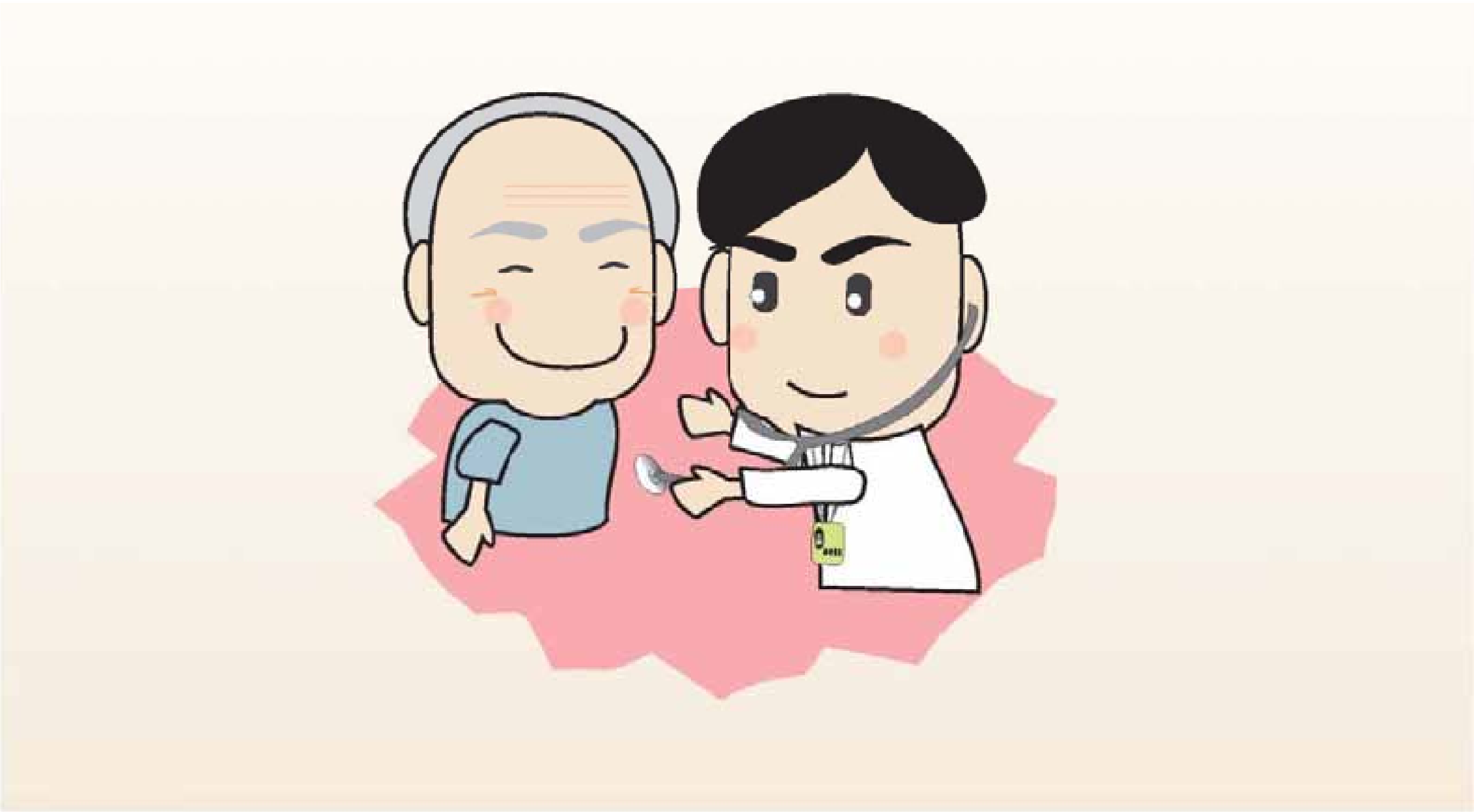

銀髮族應該做那些健康檢查呢?

建議每年應接受一次健康檢查，這對慢性病的檢查而言已足夠，檢查內容包括量身高、體重和血壓；也驗血和小便，檢查是否有貧血、糖尿病、高血脂或高尿酸、肝功能與腎功能等，醫師也會在瞭解您的生活習慣和過去生病情況後，給您適當的保健建議。

當然，也可以請教家庭醫師，根據健康狀況、健康危險因素及費用預算等，規劃更適合您個人的健康檢查。

健康銀髮族的基本健康檢查項目及檢查頻率

項目	頻率
身高、體重、視力、口腔	每年
血壓	
血糖	
血脂肪	
糞便潛血檢查	
子宮頸抹片[1]	
乳房X光攝影檢查[2]	每1～2年

1.子宮頸抹片檢查，若有規律做且都正常時，70歲後可停做。

2.乳房X光攝影若有規律做且結果都正常時，75歲後可停做。

視力保健

視力功能會隨著年齡的增長，逐漸衰退，影響日常生活活動。常見的問題有白內障、青光眼、糖尿病視網膜病變及老年性黃斑部病變等眼疾，但仍可經由視力保健與眼疾篩檢，延緩衰退。

視力保健小叮嚀：

1. **定期專業眼科檢查：**

   建議65歲以上的銀髮族每年定期眼睛檢查1次，及配戴正確度數眼鏡。眼睛檢查的內容包括視力、眼壓與眼底斑(視網膜與視神經)。

2. **預防眼睛過度疲勞：**

   避免長時間近距離的工作與近距離閱讀，要有充分的休息及睡眠，及避免強光(如夏日陽光)的照射。閱讀環境選擇適當的照明、良好的紙質、清晰的印刷、大小適當字體，休息時多看遠方，避免眼力負擔。若有視力不良，需尋求專業諮詢，不宜開車或夜間單獨出門，並且要預防跌倒。

3. **自行眼睛視力檢查：**

   在看電視時，可「睜一隻眼、閉一隻眼」，看是否相差很多，提早發現單眼疾病，注視電視的畫面直線條格線是否扭曲，提早發現視網膜病變。

口腔保健

「民以食為天」，口腔是第一道關卡，保持口腔衛生及功能，才能享受美味及咀嚼的口感，擁有健康的身體。

口腔保健小叮嚀：

1. **口腔的清潔**

   刷牙、牙線使用及輔助清潔工具，如：牙間刷、口腔清淨器、電動牙刷、或舌面牙刷等使用，確保口腔清潔。必須早、晚、餐後潔牙。以兩顆牙齒為單位，振動幅度小的方式輕柔刷牙，以免牙齒磨耗受損。

2. **定期口腔檢查**

   除對牙根蛀牙及牙周病預防外，每6個月定期做洗牙結石、口腔潰瘍或口腔粘膜病變、口腔腫瘤之檢查等。

3. **食物之選擇與調整**

   老年人生理機能減弱、咀嚼欠佳、消化吸收不良，必須調整食物種類。如：減少高熱量食物；刺激性食物應避免；醃製、炸燻食物應戒除，以清淡溫和性食物為佳，不宜過酸過甜，如飲用醋，琺瑯質會

軟化流失，牙齒表面酸蝕出許多凹洞，過冷過熱牙齒都會酸痛。喝完酸性飲料，用白開水漱口，降低口中酸性再刷牙。

4. 牙刷的選擇

牙刷的選擇以使用小頭、軟毛、刷毛不要太密的牙刷較佳，每3個月更新牙刷。

5. 假牙裝配、調整或保養

需要裝配假牙，趕快裝配，如不適合，請調整或重新製作。活動假牙，睡覺前清洗後，放入裝清水容器，避免假牙因乾燥而變形。活動式假牙不要24小時都戴著，最好睡覺時不戴，以利口腔組織休息復原。戴活動假牙後，至少每6個月至1年定期回診接受檢查與調整，以確保口腔健康並避免口腔疾病的發生。

服藥須知

重視用藥安全

藥物可以讓我們生活品質更好，活得更健康，但是65歲以上的老年人在服用藥物時要更加小心，尤其是服用許多不同的藥物 。

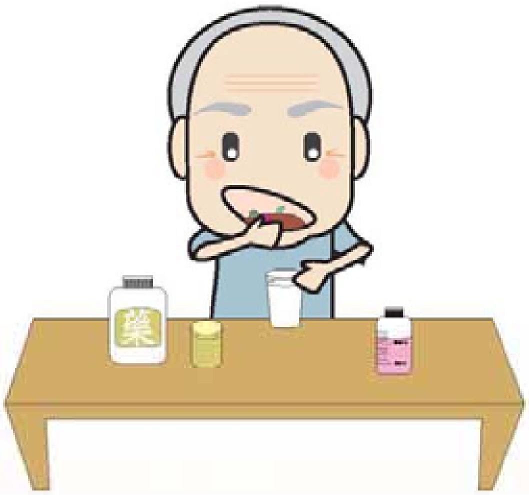

您必須知道的事

什麼是藥物 ？

- 需經由醫師看診開立處方箋所取得的藥物
- 成藥
- 綜合維他命或是健康保健食品

看診時

- 告訴醫生或護士您有服用那些藥物，如果您服用成藥，必需要讓您的醫師知道。
- 當你有過敏或是藥物導致不適(紅疹、腸胃不適、頭暈、心神不寧。)要告訴醫生和護士
- 要知道如何使用藥物，適時提出疑問。

銀髮族服藥須知

使用藥物需知道：

1. 藥名及藥物清單
2. 我要一天吃幾次
3. 飯前吃還是飯後吃
4. 使用的意義(藥物的作用)
5. 停藥的時機
6. 忘記吃藥時該怎麼辦
7. 這些藥物會有什麼副作用
8. 當有問題時可以找誰諮詢

為了用藥安全，請您一定要：

1. 按時遵從服藥(使用日曆、卡片或使用分藥盒來提醒服藥時間)
2. 不要自行減藥、停藥
3. 即使症狀相同不要服用別人的藥物
4. 藥物避免混合酒精使用
5. 藥物放在安全的地方，避免小孩拿到。

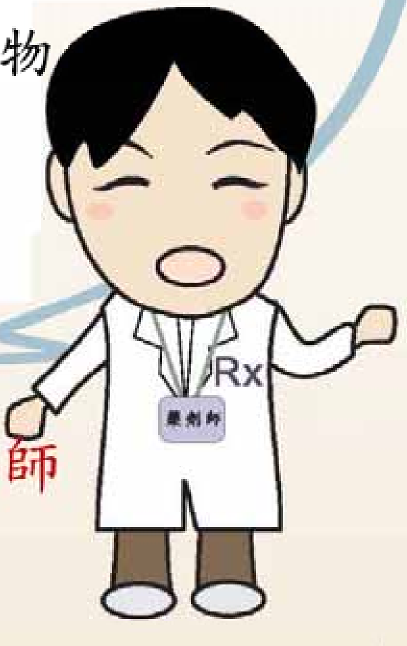

用藥有問題，請隨時諮詢醫師或藥師

預防接種小叮嚀

預防接種是減少老年人感染疾病的重要方法，目前針對65歲以上老年人，最常被建議的預防接種的項目為流行性感冒疫苗、肺炎鏈球菌疫苗及破傷風疫苗。

流感疫苗

接種時程：每年一次，每年十月份開始接種。

疫苗費用：免費提供65歲以上老年人施打。

流行性感冒的感染途徑是飛沫傳染，因此被感染者咳嗽或打噴嚏，就可能將病毒傳播出去，其中以抵抗力較差的幼童及老年人更容易被傳染。與一般感冒所產生症狀雖有部分相同，程度卻顯嚴重許多，流行性感冒除可能持續高燒3至4天外，同時還會出現痛、喉嚨痛、咳嗽、鼻塞、流鼻水、聲音沙啞等症狀。

流行性感冒的「高危險群」，主要是指罹患糖尿病、心肺疾病、中風、肺結核、塵肺症、健保重大傷病的65歲以上老人，以及罹患糖尿病、中風而居住於安養等機構的受照顧者與工作人員，接種流感疫苗可減少百分之五十至六十，因罹患流感而導致嚴重併發症機會。為照顧國民健康，政府針對65歲以上老人提供免費流感疫苗接種，可逕洽當地衛生所查詢。

肺炎鏈球菌疫苗

肺炎鏈球菌主要會引起肺炎、菌血症、腦膜炎及中耳炎等，為社區型感染的主要病菌，尤其此菌抗藥性問題日趨嚴重，是造成老人感染、住院、失能甚至死亡的重要原因。

接種時程：65歲以上接種一次。

若65歲以前施打，且超過5年以上，則建議追加一劑。

疫苗費用：自97年10月1日起，75歲以上老人免費接種。

（部分縣市免費接種年齡條件放寬，請逕洽當地衛生所查詢。）

破傷風疫苗

破傷風是由破傷風桿菌入侵傷口所致，細菌分泌外毒素造成肌肉痙攣。此疾病之致死率約在30%～60%之間，且以老人及小孩最嚴重。除了每10年追加一劑外，若發現傷口被土壤、糞便污染，或生鏽器具割傷，需於治療時告知醫師，由醫師決定是否施打疫苗。

接種時程：每10年追加一劑以維持主動免疫力。

疫苗費用：自費接種。

高血壓

高血壓是什麼?

血壓是血液在血管流動時對血管壁產生的壓力，正常血壓為收縮壓低於120mmHg及舒張壓低於80mmHg，而收縮壓等於或超過140mmHg或舒張壓等於或超過90mmHg，便可能是高血壓，不過須安靜坐著休息5分鐘後再重量一次血壓，或在不同場合或時間再量，才能確定是否真有高血壓。

表、血壓的分類標準

血壓分類	收縮壓(mmHg)		舒張壓(mmHg)
正常	＜ 120	和	＜ 80
高血壓前期	120～139	或	80～89
第1期高血壓(輕度)	140～159	或	90～99
第2期高血壓(中、重度)	≧ 160	或	≧ 100

量血壓注意事項：

1. 測量前先將手臂上的衣物脫下，休息5分鐘。
2. 血壓計、手臂與心臟齊高，手掌向上。
3. 測量時雙手均分別測量，以較高值為準。
4. 在飲酒、運動及沐浴後30分鐘內不要測量。

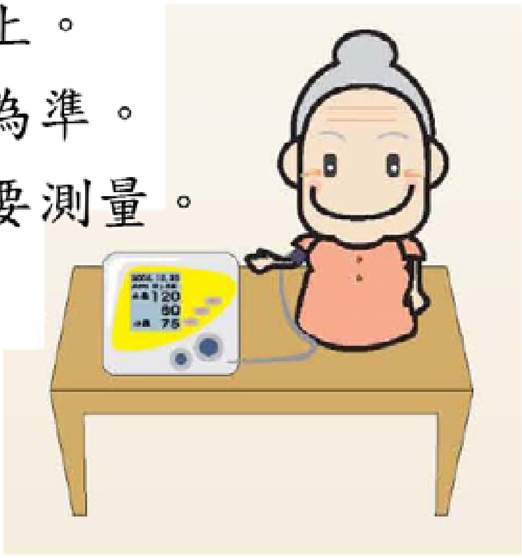

血壓應該如何控制?

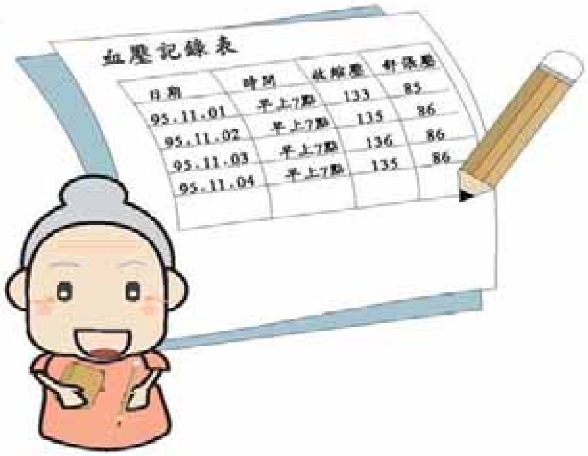

大部分高血壓的狀況，都可以藉由飲食、生活習慣的改變，或者是規律的服藥而得到有效控制。

飲食和生活方式的調整

1. 飲食方面：多吃蔬菜及水果、減少攝取高膽固醇及高鹽份的食物。
2. 運動方面：請參考本手冊「運動休閒生活」篇(第12頁)來運動。合併有心臟病或肺病者，須先與醫師討論運動的安全性。
3. 日常生活方面：須維持理想體重、睡眠充足、適度紓解壓力及保持輕鬆心情、避免吸菸及過量飲酒。

遵照醫師指示服藥及追蹤血壓

聽從醫師的指示每天服用降血壓藥，不可以只在不舒服或量到血壓高時才服藥，即使血壓已經控制在正常的範圍，仍須依照醫師指示服藥，不可自行停藥或減少藥量。

最好能養成定時及不舒服時量血壓並記錄的好習慣；最好家裡備有血壓計，可以自己測量、請家人幫忙量血壓或到附近醫療院所量，看診時可以將血壓紀錄帶去供醫師參考。

高血脂

高血脂是什麼?

血脂就是血中所含的脂肪，高血脂就是我們常聽到的「膽固醇過高」、「血濁」、或是「血油過高」，缺血性心臟病、腦中風、周邊血管疾病及腎臟疾病等動脈硬化疾病都與高血脂有密切的關係。銀髮族可在晚上空腹8小時後之隔天早上接受血脂肪檢查，項目包括總膽固醇、三酸甘油酯、高密度脂蛋白與低密度脂蛋白等，如有任何一項超過標準時(如下表)，便應注意飲食調整及體重控制。

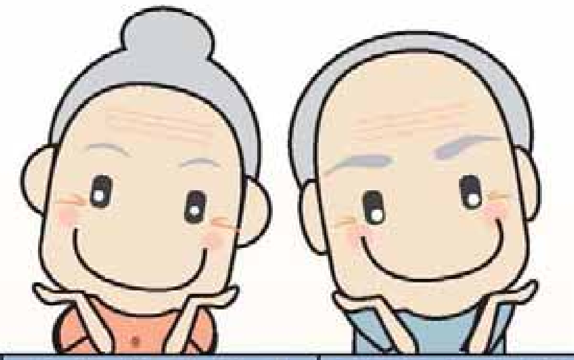

表、血脂肪的診斷標準

	總膽固醇	三酸甘油脂	低密度脂蛋白(不好)膽固醇	高密度脂蛋白(好)膽固醇
正常值	＜ 200	＜ 150	＜ 100	♀女性 ＞ 50<br>♂男性 ＞ 40
邊際高值	200～239	150～199	130～159	–
過高值	≧ 240	≧ 200	≧ 160	–
過低值	–	–	–	過低值 ＜ 40

(2001年美國膽固醇教育計畫成人治療指引-第3版)

*單位為mg/dl；若高密度膽固醇≧60mg/dl，對缺血性心臟病有保護作用。

不用藥物可以治療高血脂嗎?

健康飲食、多運動、不飲酒、不吸菸和控制體重都可以有效降低血脂肪。

飲食控制

1. 飲食烹調宜減少油炸改用清蒸、水煮或其他方式替代，少勾芡及少用糖，另應多選用纖維較多的蔬菜、水果、燕麥、黃豆、麥片、豆莢及全穀類食物。
2. 炒菜時亦儘量選用不飽和脂肪酸較高之植物油，如橄欖油、菜籽油、沙拉油、花生油等，不要用豬、牛、雞等動物性油。
3. 避免食用內臟、烏魚子、蟹黃或蛋黃等高膽固醇食物，食用肉類時先把肥肉或皮去除。
4. 少喝酥皮、奶油等濃湯或勾芡湯，不宜多吃蛋糕、餅乾、精緻小西點、乳瑪琳及酥炸食品等高糖高熱量之食物。
5. 飲酒要有節制。

運動控制

運動可以維持理想體重，避免肥胖，增加心臟及肺臟的功能，同時也可以降低血中膽固醇、三酸甘油酯、低密度脂蛋白並增加高密度脂蛋白，增加血管彈性及血液循環。

高血脂的藥物治療

接受飲食計畫3-6個月、控制體重、運動及戒菸後，若血中膽固醇及三酸甘油酯濃度仍偏高，此時醫師就會給予藥物治療。如果經醫師診斷需服用降血脂藥物時，請不要任意停藥，否則可能會造成血中膽固醇再度上升。如果症狀改善，應由醫師再做一次血脂肪檢驗，醫師將會視您的情況決定是否需減量或繼續服藥。

成人血脂控制目標		一般成人	心血管或糖尿病者
	總膽固醇	< 200	< 160
	三酸甘油酯	< 150	< 150
	高密度脂蛋白	男> 40、女>50	
	低密度脂蛋白	< 130	< 100

糖尿病

糖尿病是什麼疾病?

糖尿病是因為胰臟的胰島素分泌不夠或功能不好所造成，導致自腸道吸收至血中的糖份不易進入細胞內代謝，當空腹血糖值上升至超過126mg/dl時，即有可能是糖尿病患者。典型的徵狀為吃多、喝多、尿多及體重減少，但大多數病人不會有明顯症狀，故必須從驗血才能發現糖尿病。

如何預防糖尿病?

維持健康的生活型態是非常重要的，包括定期檢查血糖、飲食、維持理想體重、規律且適當的運動。請您參考本手冊「健康生活型態」篇(5～16頁)。

有糖尿病時該怎麼辦?

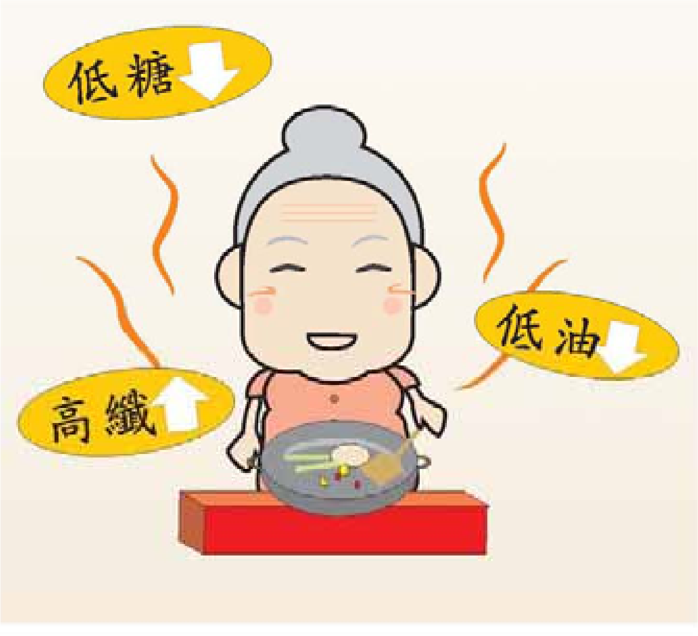

1. 飲食方面：吃八分飽，且使用低糖、低油及高纖食品。喜歡吃甜者可用代糖，零食可吃代糖調味的仙草、愛玉或各式蒟蒻。
2. 運動方面：除預防上應注意事項外
   a. 若合併有視網膜病變、神經病變、中風、心臟病或肝腎功能不好者，不適合劇烈運動，故需先與醫師討論運動之安全性。

b. 不可在空腹時去運動，更千萬不要在吃糖尿病藥後，沒吃東西便去運動。

C. 身邊應隨時準備一些方糖(或糖果)、餅乾或飲料，以便發生低血糖時可以吃。

d. 隨身攜帶「糖尿病護照」

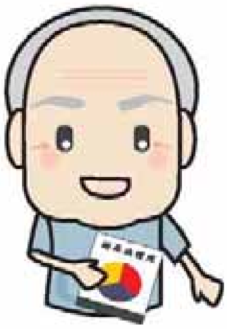

3. 若控制飲食及運動後仍不能達到理想血糖值時(如下表) ，便需接受藥物治療。
4. 需固定追蹤腎功能、血中膽固醇濃度、至眼科醫師處檢察視網膜以預防糖尿病可能引起之嚴重併發症。
5. 避免皮膚過於乾燥或擦傷、碰傷，減少皮膚感染之機率。
6. 選擇合適之鞋襪，同時每天檢查足部是否有傷口或過度壓迫之紅腫，避免造成足部之傷害。
7. 必要時需以藥物將低密度膽固醇控制在100mg/dl以下，膽固醇在200mg/dl以下及三酸甘油脂在150mg/dl以下。
8. 特別的叮嚀：不吸菸且不過量飲酒。
9. 必要時需將血壓控制在< 130/80 mmHg，或遵照醫師指示使用阿斯匹靈藥物，以預防心血管疾病。

表、血糖正常值、糖尿病診斷標準與治療目標值

	血糖正常值	糖尿病診斷標準	治療目標值
空腹血糖	80～99	≧ 126	70～130
飯後2小時血糖	＜ 140	≧ 200	140～180
糖化血色素*	＜ 6%	－	＜ 7%

血糖值單位為：mg/dl　*為了解最近三個月平均血糖控制情形的指標

腦中風

腦中風是什麼?

腦中風分為兩種類型：

1. 出血性：腦內血管破裂出血所致
2. 缺血性：腦內血管阻塞所致

腦中風有什麼症狀

1. 突然發生的單側臉、手或是腳的無力或是麻木感。
2. 急性的意識混亂或是無法與人溝通
3. 單眼或是雙眼忽然看不清
4. 突發的暈眩、失去平衡或是走路困難
5. 突發的不明原因頭痛

有以上的症狀，請儘速就醫。

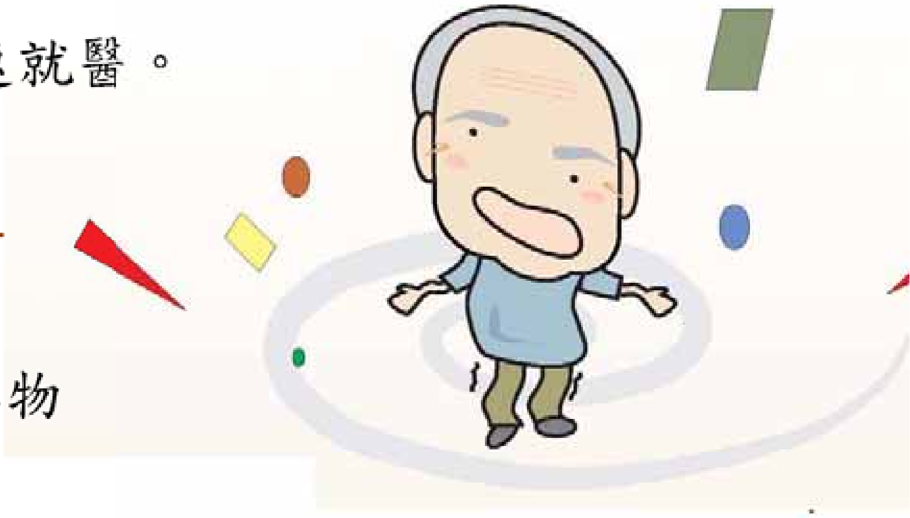

如何預防腦中風?

1. 維持適度的體重
2. 避免升高血壓的藥物
3. 降低鹽分攝取
4. 多攝取蔬果，增加由食物攝取的鉀(如香蕉、柳丁)
5. 增加運動量
6. 戒菸節酒

發生腦中風時該怎麼辦?

1. 先躺下來，若有嘔吐現象，便應側躺，避免嘔吐物及分泌物掉入肺部，以避免發生吸入性肺炎的危險。
2. 若意識不清時，不要給他(她)吃東西或餵藥。
3. 一定要儘速就醫，因就醫時間是影響治療的關鍵性因素，能越早治療，治療效果可能越好。
4. 當病程穩定後，遵醫囑服藥，以控制好血壓、血糖、血脂肪並接受復健治療。

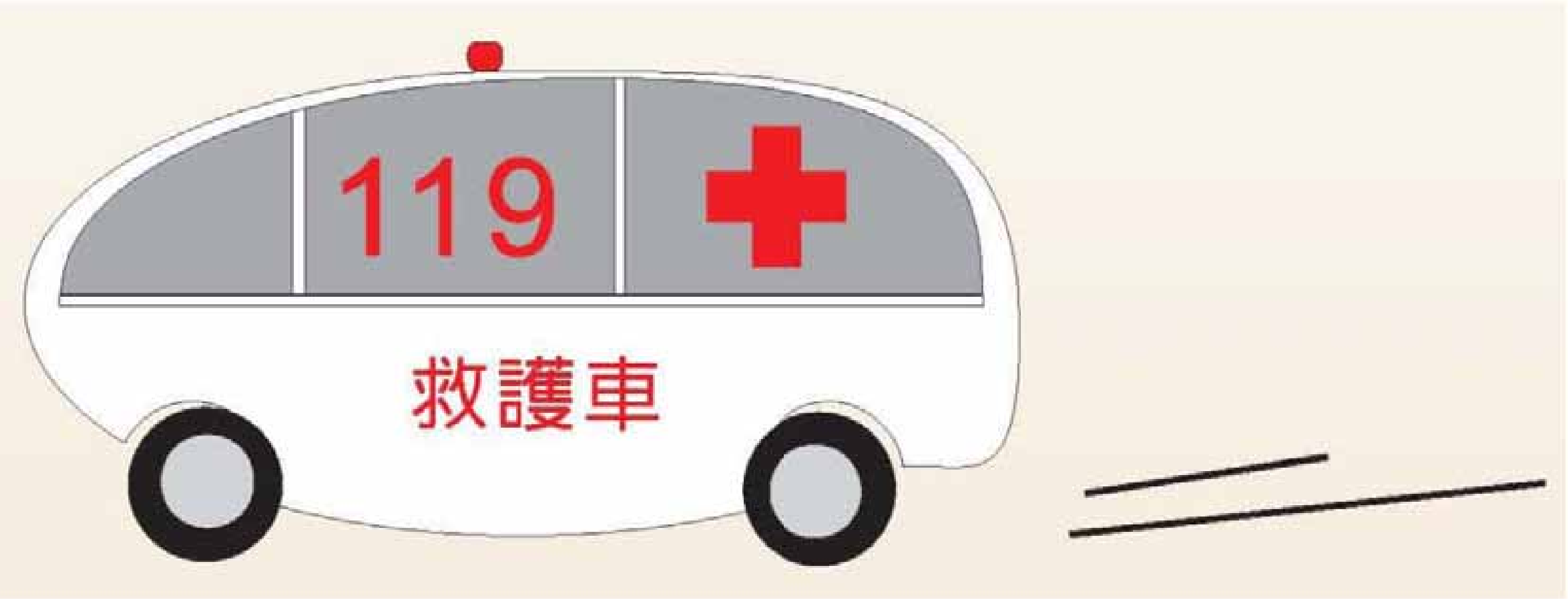

退化性關節炎

退化性關節炎是什麼?

退化性關節炎是常見的關節疾病，主要病因為老化、體重過重、活動過量或受傷等，好發年齡為50歲以上且以女性較男性多見。部位以膝蓋、頸椎、腰椎或手指為主；症狀為關節僵硬、疼痛或活動困難;嚴重時會壓迫到神經，引起手腳痲痺或無力。

如何預防退化性關節炎?

保護關節

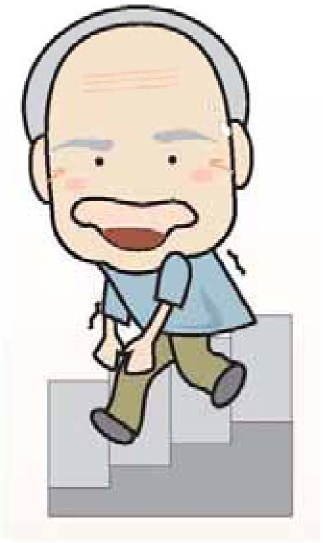

減輕關節受力最重要，例如減輕體重、減少上下樓梯或爬山、走動或站立的時間不要太久，及盡量不要用跪、盤腳或蹲著的姿勢。

增強關節附近肌肉的力量

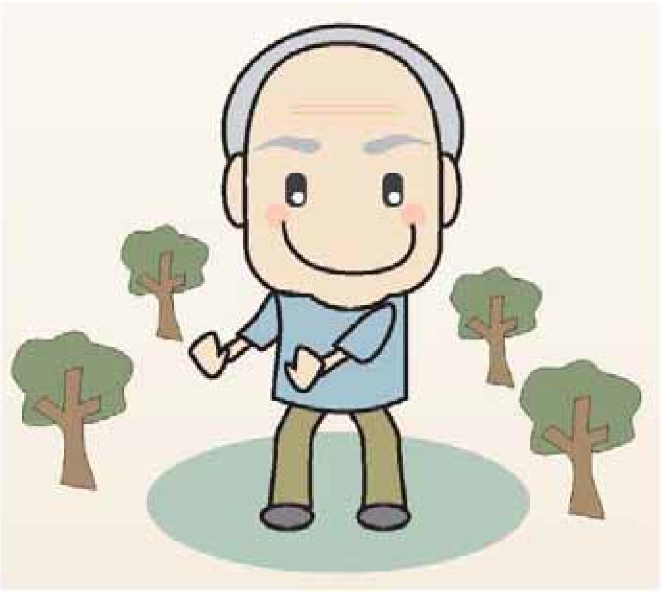

至少每天有一次約30分鐘的適度運動，如健走，以增強腿部肌力，嚴重者宜考慮、作水中運動如游泳；另太極拳可加強平衡感。

有退化性關節炎時該怎麼辦?

平時要減輕關節負擔

　　不舒服時最重要是要讓關節休息，必要時可用拐杖分擔壓力。

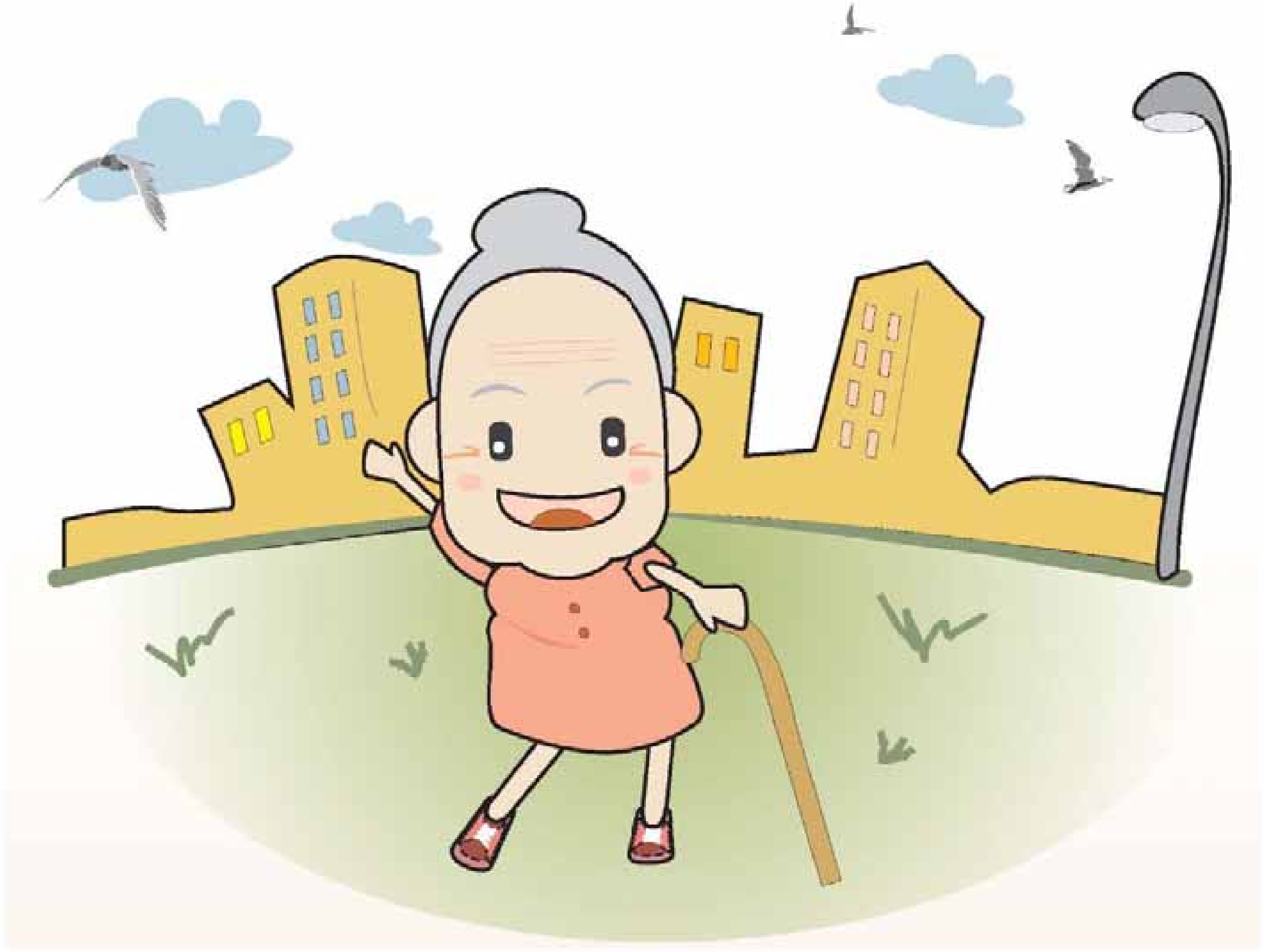

遵醫囑使用止痛劑(消炎藥)

　　銀髮族服用止痛藥後易發生胃腸潰瘍或腳水腫，故疼痛時建議先休息或選用外敷藥，如薄荷油、辣椒膏或消炎貼片等。效果不好時，請找醫師診斷，再決定是否須服用止痛藥。

骨質疏鬆症

骨質疏鬆症是什麼?

骨質疏鬆是骨質流失過多，可經由骨質密度檢查的結果得知(如下表) ，發生的主要原因為老化、停經、缺少運動及長期臥床等，女性較男性早發生且較為嚴重，部位以脊椎、大腿骨、手腕骨及上臂近肩膀骨頭為主，通常無症狀，但容易因搬重物、咳嗽或跌倒而導致骨折並引起疼痛，嚴重時將會影響生活品質和威脅生命。

骨密度值 (T值)	正常	骨質不足	骨質疏鬆
	＞-1.0	-1.0～-2.5	＜-2.5

如何預防及早期治療骨質疏鬆症?

增加鈣質攝食以保存骨本

多吃含鈣食物，如深色蔬菜、豆腐及牛奶；每天運動、曬太陽10分鐘、不吸菸、不飲酒；並建議每日補充約1000～1500毫克的鈣片及400～800國際單位(IU)的維他命D。

有藥物治療可選擇

有藥物可增加骨質密度或減慢骨質流失的速度，但需先經醫師評估是否適當。

有骨質疏鬆症時該怎麼辦?
預防跌倒最重要

身體保健與治療

每年做一次健康檢查，請家庭醫師幫您看是否有服用可能引起跌倒的藥物(如降血壓藥、良性攝護腺肥大用藥)；另需自己戴合適的眼鏡及治療眼疾、控制血壓、血糖和體重。

注意環境安全

在浴室牆壁安裝握把、地面加裝防滑墊及保持乾燥；也要清理走道的雜物、穿合適大小的鞋、臥室內裝有夜燈、樓梯處需有足夠照明等。

維護肌力、保持平衡

適度運動，如做抬腿運動及平衡訓練，並避免姿勢改變過快，起床後先坐在床邊35分鐘後才站起來；必要時使用高度適中及有橡膠頭的拐杖。

避免重心不穩的危險動作

不要過度向前或後彎腰、避免快速扭腰轉身或手高舉過頭，不能提重物、不要站到椅子上拿東西或爬不穩固的樓梯等。

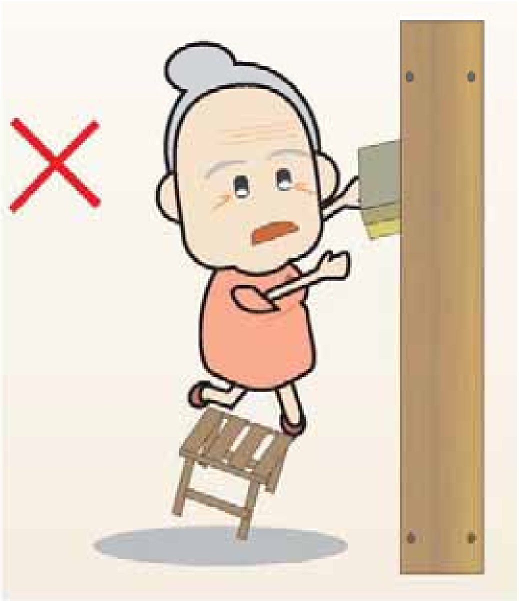

跌倒

銀髮族跌倒的嚴重性

事故傷害是台灣老年人的第七大死因，其中一半以上與跌倒有關。因老年人的骨質密度不足、皮膚變薄、血管硬化等，雖然可能只是輕輕的摔一下，亦可能引起嚴重的出血或骨折，也較易發生合併症甚至死亡。跌倒後的傷害，可能會造成行動上或自我生活照顧上的困難，從此需要家人照顧，對生活品質和家人照顧壓力都有很大的衝擊。

銀髮族跌倒的原因

個人因素：視力、聽力、平衡感及肌力變差，患有心律不整、腦中風、巴金森氏症或關節炎等疾病，使用鎮定助眠藥物或同時合併多種藥物時，容易出現站不穩現象而導致跌倒。

環境或物件因素：如家裡雜物多、屋內光線太暗、地板溼滑、拖鞋大小不合適或鞋底光滑等，易因碰撞到物件而跌倒或滑倒。

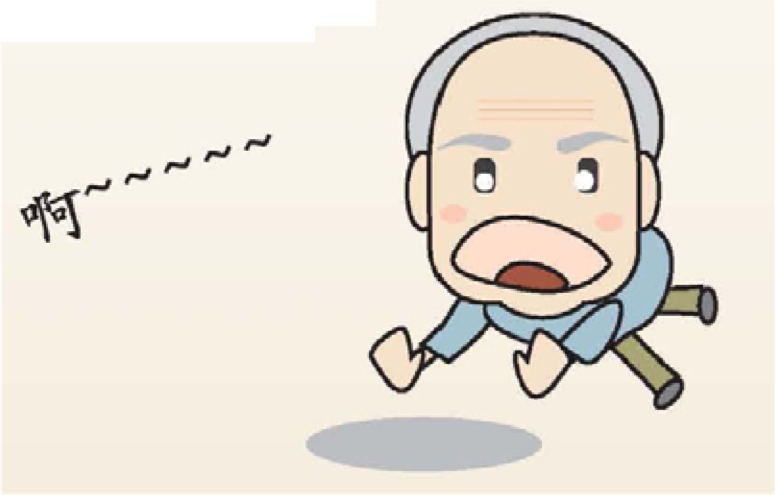

如何預防跌倒?

定期評估健康狀況

就醫時應告知醫師過去的疾病、檢查結果及使用的藥物。定期檢查視力及聽力，配戴適合的眼鏡、助聽器，同時由醫師評估使用之藥物是否容易增加跌倒之風險、身體狀況是否適合運動等項目。

規律適度運動

規律及適度的運動可增強肌力、柔軟度和平衡感以預防跌倒。運動種類的選擇需配合自己的身體狀況，同時也要選擇輕便舒適又吸汗的穿著，加強運動前後之暖身及緩和運動，選擇通風良好、開闊且無障礙物之場地，穿著適當之鞋襪，正確使用拐杖、助行器或輪椅等輔具等，使運動時更為安全。

營造安全居家環境

光線充足、臥室至廁所之走道應留一盞夜燈，家具擺設宜簡單且位置應固定。電線應收好或固定在角落；樓梯、走道、馬桶邊及浴盆旁應安裝扶手；浴室地板應有防滑墊；地面不要打蠟，若地上有水或打翻湯水時，應立即擦乾。

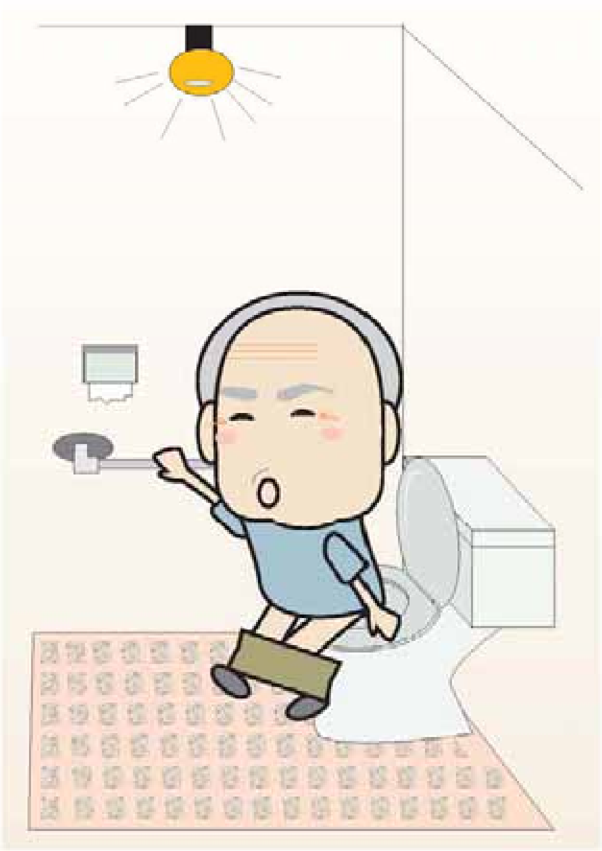

長期失眠

長期失眠是什麼?

失眠是指難以入眠、睡著後又容易醒來、過早醒來後便不易再入睡或早上睡醒時仍覺得疲倦和沒有精神。短期的失眠主要與生活壓力、有煩惱的事情或環境改變有關；若失眠持續超過3個星期以上，便是長期失眠了，這時候應去看醫師，讓醫師替您找出可能的原因和解決失眠的問題。

您必須知道的事

有時銀髮族以為自己有失眠，但其實可能只是睡眠型態與年輕時不同，不宜過度擔心有失眠，也未必一定要睡滿8小時，只要不要影響到日常生活就表示睡眠是足夠的，以免增加心理負擔。睡眠型態的改變，主要是熟睡期和深睡期減少、淺睡期增加和不容易入睡等。

常見造成長期失眠的原因中，以焦慮和憂鬱心情最常見，但身體不舒服如夜間咳嗽、關節酸痛、心臟衰弱或巴金森氏症等疾病、或吃了會影響睡眠的藥物如百憂解或含類固醇的止痛藥等也很常見。

有長期失眠時該怎麼辦?

若經過醫師詳細評估後，發現無特殊影響睡眠的原因時，治療上是以調整睡眠衛生習慣(即非藥物療法)為主，必要時再考慮使用藥物。

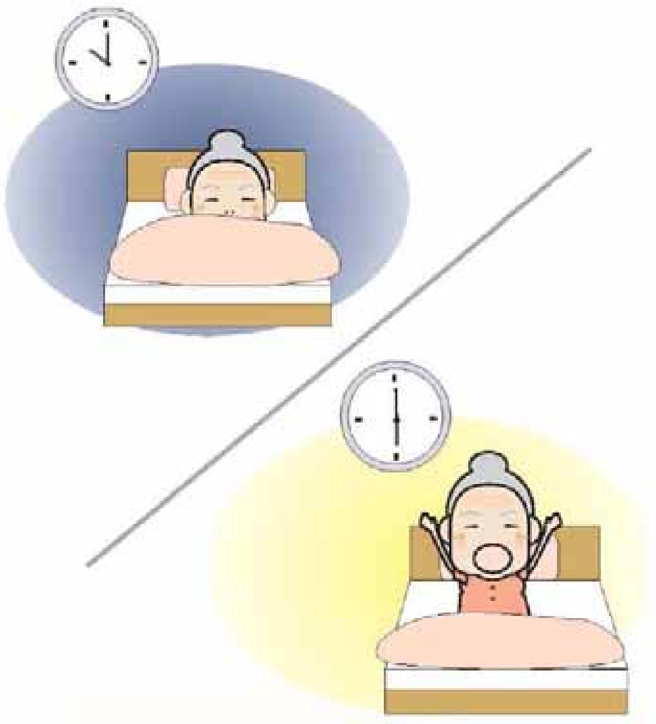

睡眠衛生習慣(非藥物療法)

1. 固定就寢及起床的時間。
2. 晚餐後避免飲茶、咖啡、吸菸或飲酒。
3. 維持舒適安靜的睡眠環境。
4. 不要在臥房內看電視或看書。
5. 若躺床上超過30分鐘仍睡不著時，可起床到別的房間做些靜態活動，如看書或燙衣服等，當感到想睡時再躺回床上睡。
6. 白天最好有規律運動，在睡前則可做幫助放鬆之活動如柔軟操等。

藥物治療

若非藥物療法效果不佳時，醫師會建議短期使用安眠藥物，請遵醫囑服用。長期使用時，可能會產生依賴性或增加跌倒的危險，故不宜長期使用。

打鼾

為何會打鼾

隨著年紀增長、或體重增加，喉嚨中許多組織變得較鬆弛及肥厚。睡眠時因身體放鬆，呼吸道就容易變得窄小而導致鼾聲。

多少人會打軒

「打鼾」在銀髮族身上十分常見，比率可高達四到六成以上。

打鼾的影響

打鼾一則困擾枕邊人，再者，因為怕尷尬，而影響社交活動。而且根據研究，打鼾者較常有高血壓、心臟病等。更嚴重者，它可能合併有阻塞型睡眠呼吸中止症(一種在睡眠中會發生數十次至數百次呼吸暫停及缺氧的疾病)。而睡眠呼吸中止症又會再引發高血壓、冠狀動脈疾病、心肌梗塞及中風等。

何時該看醫師

經常打鼾且合併白天精神不振、嗜睡、或合併有高血壓或心血管疾病者，都應找相關醫師諮詢、檢查。

打鼾如何處理

許多醫療機構都設有打鼾(或睡眠呼吸)門診，可協助民眾診斷(睡眠呼吸檢查)及治療如減重、戴牙套、手術、或使用睡眠陽壓呼吸輔助器等)。只要接受有效治療，這些健康的危害都可消除。

尿失禁

老年人失禁的導因

失禁是老人常見的問題，卻不是老化的必然現象。老年人失禁的導因包括：

1. 膀胱肌肉無力
2. 膀胱肌肉不自主的收縮
3. 神經系統的疾病所導致的膀胱控制問題(中風、帕金森氏症)
4. 身體活動受限(如：關節炎)導致解尿不便
5. 男性前列腺肥大
6. 骨盆底肌肉鬆弛

尿失禁的種類

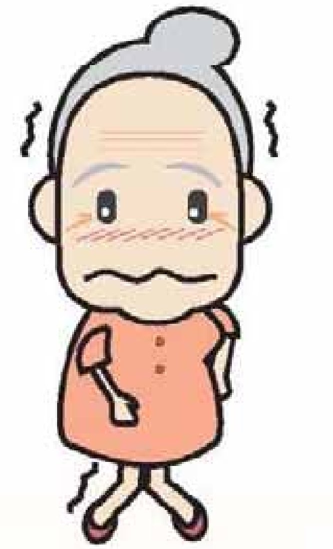

尿失禁的種類可以分為下列幾種：

1. 應力型尿失禁：打噴嚏、咳嗽和腹部用力時會漏尿。
2. 急迫型尿失禁：尿急會來不及上廁所，就尿出來。
3. 滿溢型尿失禁：膀胱無法順利排尿，尿液太滿而溢尿。
4. 功能型尿失禁：由於日常生活能力受限，所以無法及時解尿而漏尿。

如何改善尿失禁

改善尿失禁在老人族群最常見的方法有下列幾種：

1. **骨盆底肌肉運動：**

運動骨盆底肌肉來增加禁尿的能力

a. 通常的執行方式包含收縮骨盆底肌肉數到三，然後再放鬆數到三。運動目標在於一天做三次一次做至少十下。

b. 如果覺得無法夾緊骨盆底肌肉，可以尋求失禁運動門診協助，可以使用生理回饋儀的協助。

2. **定期排尿訓練：**

藉由定期解尿的方式，來建立膀胱解尿的常規。這樣的訓練結合骨盆底肌肉運動可以有效的緩解急迫型與滿溢型尿失禁。

3. **生活型態改變：**

包含減重、戒菸、節酒、節制咖啡因的攝取、避免便秘與提重物都可以有效的緩解尿失禁的病程喔!

4. 如果以上的策略都無法有效緩解失禁，可以尋求專科醫師的協助，醫師處理尿失禁的方式包含：藥物治療、局部注射藥物、輔助用品的使用與手術等。

最後，失禁雖然是老人常見的問題，但透過有效的行為改善、運動或是醫師的協助，還是可以有效解決溼答答的困擾，千萬不要拖延了治療的良機喔。

失智症

失智症是什麼?

失智症並不是單指記憶力變差，而是指腦部功能退化，造成對「人、時、地」都失去認知和判斷能力，最後發生日常生活活動功能喪失之慢性疾病。年齡愈大，發生的可能性就愈高，在85歲以上的銀髮族約有一半患有輕度的廣義失智症。

您必須知道的事

1. 認知功能不一定會隨年歲增加而降低。銀髮族可能比較健忘或學習新事物的能力降低，但失智症絕對不是正常老化的一部分。
2. 每年找醫師做一次認知功能篩檢。
3. 積極控制高血壓、糖尿病、高血脂及不吸菸等心血管危險因子，可有助預防血管性的失智症。
4. 多動腦，如下棋、學新知識、多跟朋友聯絡、旅遊及經常運動等，對保持腦力有幫忙。
5. 吃銀杏、女性荷爾蒙、消炎止痛藥、抗氧化劑如維他命E，是否可預防失智症?因臨床證據尚不足，暫時不建議使用。

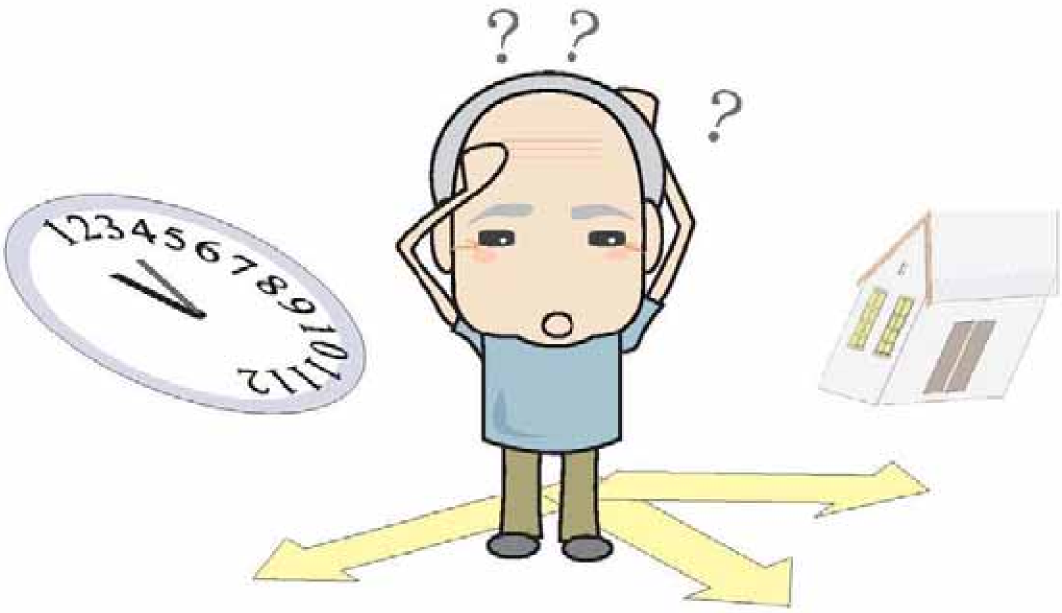

有失智症時該怎麼辦?

1.若自己或家人發覺記憶力、判斷力、理解力或表達能力有改變；對時間、空間概念不佳；情緒起伏、性情大變、自理日常生活活動的能力變差時，請儘速就醫。

2.大部分的失智症是無法治癒，現有的藥物只能控制症狀，但不一定能延緩病情，因此，要有接受無法治癒的心理準備。

3.失智症的照護需要病人、家屬與醫護人員等團隊成員共同合作。目標在於維持最佳的生活功能、增進生活品質、改善認知功能、心情及控制行為問題。

4.照顧失智症病人是長期耗體力及精神的工作，因此，對照顧病人的家屬，要不斷給予心理支持及生活輔導或支援等，以減輕其照顧壓力。

老人住宅

「老居」的發展趨勢

國人一向安土重遷，認為沒有房產即缺乏安全感，不過以後的「居」，可能是另一種型式，近十年來，台灣老人的居住狀況，與子女同住的比例不斷下降，越來越偏向老年夫妻同住或獨居的情形，甚至逐漸發展成健康老人群居的情形，因此，老人群居是未來的趨勢。

老人住宅是什麼?

針對身體狀況良好，希望保有自我空間，且能和子女維持「有點黏又不太黏」關係的銀髮族，所興建的照顧住宅及社區。最大的特色，就是有完善的安全環境及保護措施、合格的照顧人員、休閒、室內活動、健康管理及餐飲照顧等，例如養生村或仁愛之家等。

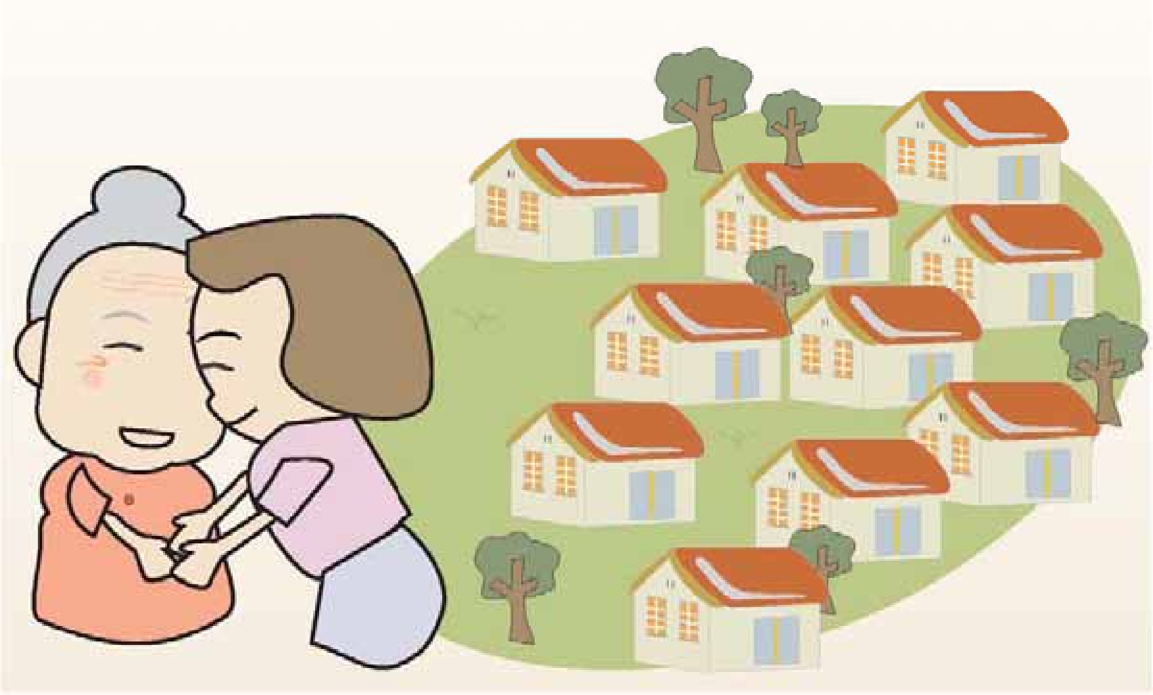

您要如何選擇老人住宅?

選擇老人住宅時，可以思考下列幾個問題：

1.入住的原因及動機為何?

2.經費預算考量?

3.由內政部或各縣市社會局所整理的資料，挑選出合適自己居住的機構。

4.現場參觀及試住體驗，並觀察以下重點：

a. 醫療、安養、休閒及居住需求設施及服務。

b. 生活環境與安全問題。

c. 交通的方便性。

5.檢討及確認契約書內容與進住條件。

理想的老人住宅，要能使銀髮族在身體活動及心智認知等方面，都能繼續學習、發展並享受健康有樂趣的生活。

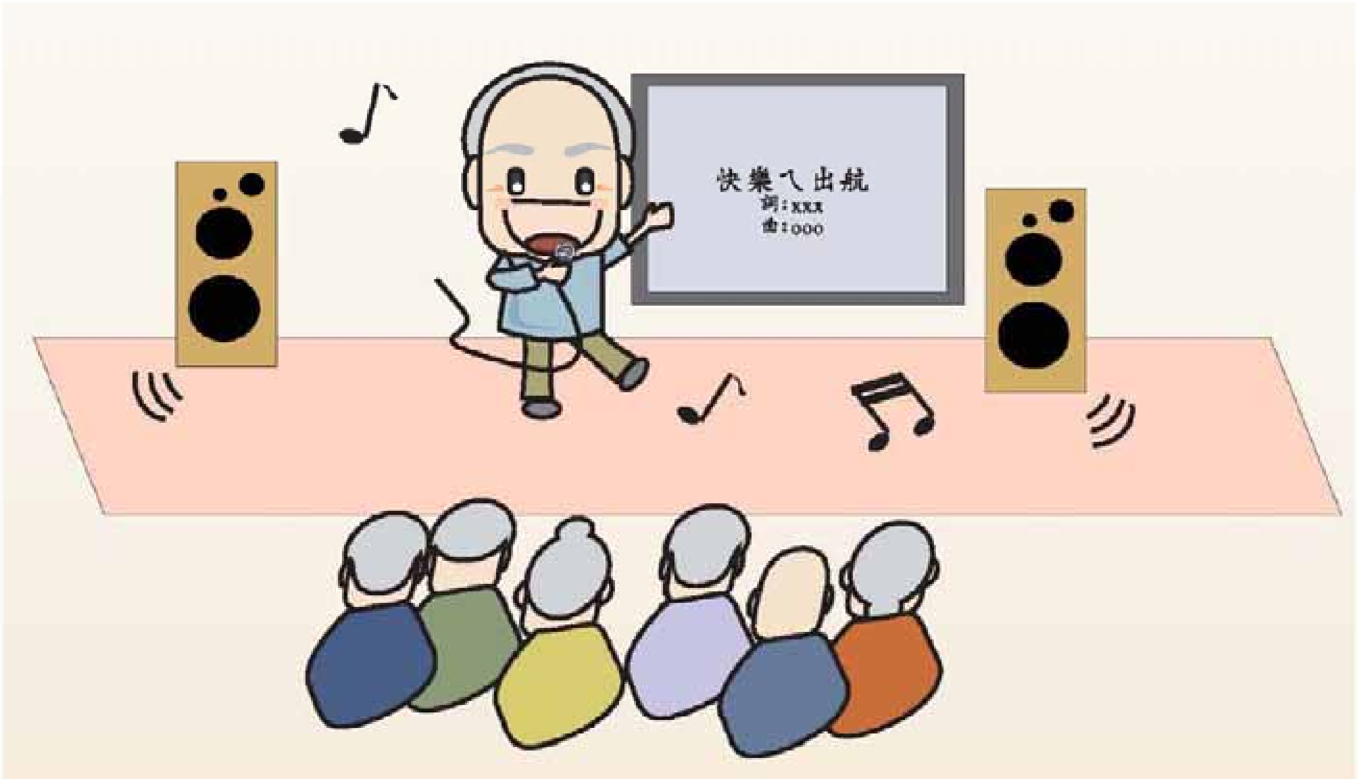

社會參與

因為社會結構變遷，人口老化，少子化及子女外出就業，老年生活孤寂、無伴，是任何人無法避免的過程，及早準備，才能活出健康、快樂生活。

瑞士UMEBA大學研究：喜歡參與社交、文化及福利社團活動者，較長壽，能從活動中找到各種解決生活問題的方法，增強免疫功能，不易生病，促進人際關係，有歸屬感，消除孤獨感，增進生活情趣，有很大助益。所以我們鼓勵銀髮族，走向群眾，多參與有益身心活動。

要成為一個快樂的銀髮族，必須擁有下列特質：

隨　　和：對大小事情，不拘束、不堅持，態度隨和。

積極樂觀：正向面對大小事情，不自怨自艾。

善用社會資源：善用社會資源，參與社區活動，提升自我價值，增進身、心、靈健康成長，如：社區發展協會關懷據點、老人會、婦女會、社區大學、長青學苑等，有助於提升銀髮族生活品質。

年歲愈大愈需要依賴外界的資源，常懷一顆喜悅、樂觀、感恩、善用外界資源的心，不僅活得快樂，同時也是最佳的抗老化良方。

機構式照護

機構式照護是什麼?

機構式照護，包含安養養護機構及護理之家二大類，一般而言，較穩定的慢性病療養或單純的日常生活照顧，屬安養養護機構的範圍；而需要較多醫療服務及技術性護理照護，則屬護理之家的範圍。

機構式照護的迷思

由於人口高齡化、少子化及小家庭的社會和婦女就業比率提升，老人獨居或需要安養者之增加，已成為必然的趨勢。內政部調查發現，近年來有意願選擇機構式照顧的銀髮族之比率有增加趨勢。因此，不宜將銀髮族接受機構照護，與傳統反哺孝親、三代同堂，或養兒防老的觀念糾結在一起，而認為子女不孝順。

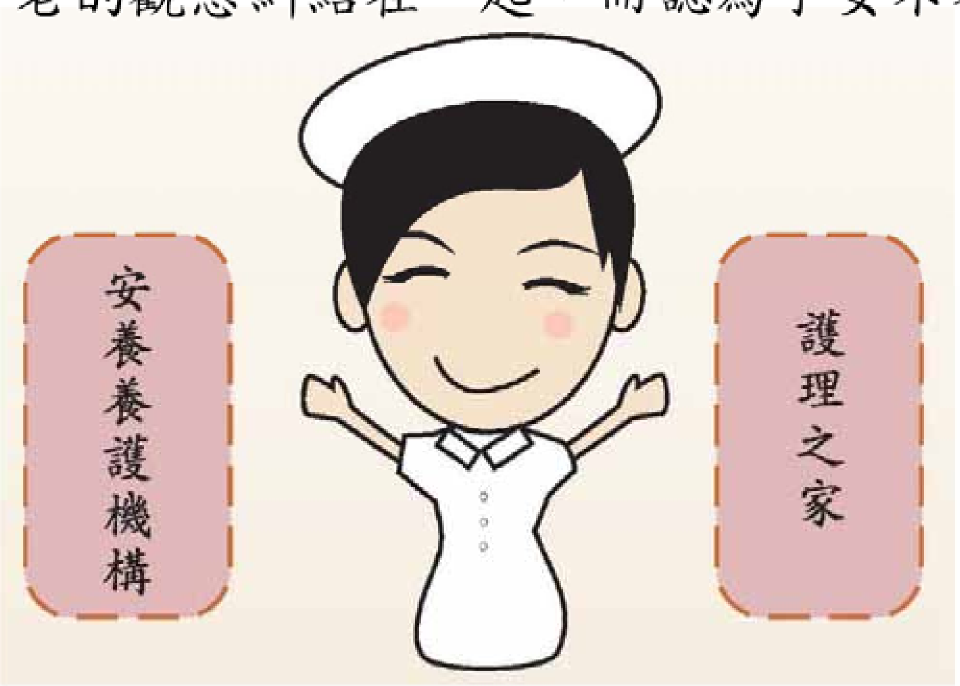

您要如何選擇機構式照護?

銀髮族需要機構式照護時，可以思考下列幾個問題：

1. 所需的照顧內容為何?
2. 對接受機構照顧的意願及感受為何?
3. 家人自行照顧失能銀髮族在人力、能力及物力上能否負擔?
4. 其他替代方案，如日間照顧、居家服務之可行性?
5. 請教專業人員的意見，不要道聽塗說。
6. 多次不同時段或臨時親自到機構參觀、評估，並多方收集資料。

選擇機構時，宜秉持貨比三家不吃虧，一分錢一分貨的理念，才能為銀髮族找到具專業品質及收費合理的機構，而不是只單純看收費價格來決定。

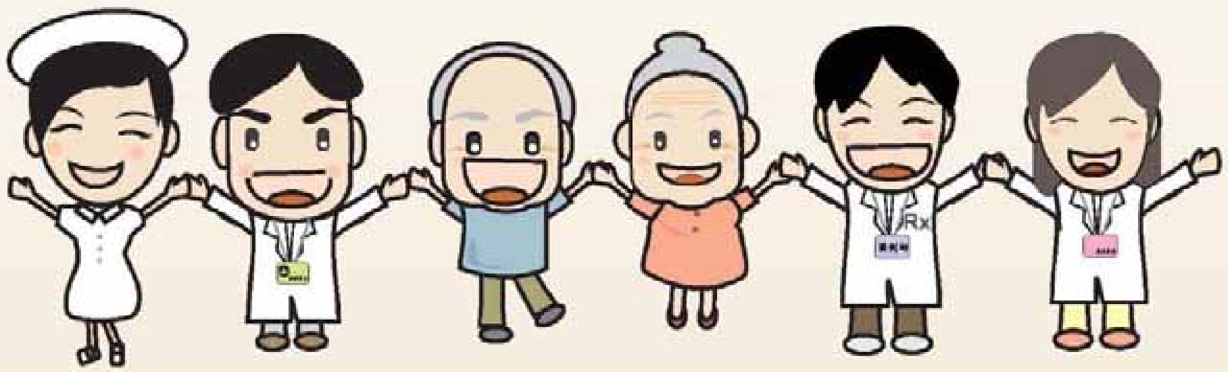

老人照護資訊

政府部分	行政院衛生署國民健康局　健康九九衛生教育網<br>http://www.health99.doh.gov.tw/　(04)2255-0177
	行政院衛生署護理與健康照護處　(049)233-2161<br>http://www.doh.gov.tw/CHT2006/DM/DM2_p01.aspx?class_no=21&now_fod_list_no=6571&level_no=1&doc_no=39350
	在家上網看醫生【台灣e學院】http://fms.doh.gov.tw/
	中央健康保險局-民眾服務<br>http://www.nhi.gov.tw　0800-212369、0800-030598
	內政部社會司老人福利服務　(02)2356-6225<br>http://sowf.moi.gov.tw/04/new04.asp
	各縣市政府社會局<br>http://www.hearingaid.com.tw/Social/social-8.htm
	全國各縣市「長期照顧管理中心」聯絡資料<br>http://www.chunda.com.tw/download/各縣市長期照護管理中心.doc
	教育部樂齡學習網<br>https://moe.senioredu.moe.gov.tw/front/bin/home.phtml
	輔具資源入口網　(02)2874-3415、(02)2874-3416<br>http://repat.moi.gov.tw/

失智症相關諮詢機構	社團法人台灣失智症協會 (02)3365-2826<br>www.tada2002.org.tw 關懷專線：0800-474-580
	天主教康泰醫療教育基金會 (02)2365-7780#14<br>www.kungtai.org.tw
	財團法人天主教失智老人社會福利基金會<br>www.cfad.org.tw (02)2332-0992
	中華民國失智症者照顧協會 (04)2473-1619<br>http://www.cdca.org.tw

其他	中華民國老人福利推動聯盟 (02)2592-7999<br>http://www.oldpeople.org.tw
	中華民國長期照顧專業協會 (02)2369-0347<br>http://www.1tcpa.org.tw
	社團法人中華民國家庭照顧者關懷總會<br>TEL：(02)2511-1751 關懷專線：0800-580-097<br>http://www.familycare.org.tw/fcgnew/
	老人諮詢服務中心：0800-22-8585<br>北區(02)2219-2528 http://www.wretch.cc/blog/cfad73990893/37922<br>中區(04)2297-5930 http://old.lkk.org.tw<br>南區(07)224-2083 http://oldfriend.joseph.org.tw
	各縣市老人服務中心(長青大學、銀髮社團、保健服務、獨居及失能老人照顧、居家服務、日間照顧等）
	銀髮族健康天地 http://www.sfit.org.tw/health/
	國家網路醫院 http://www.kingnet.com.tw/
	身心障礙者服務資訊網-各縣市身心障礙福利服務中心<br>http://disable.yam.com/consult/serve.htm

健康老化銀髮族保健手冊

編著：行政院衛生署國民健康局

編輯：台灣老年醫學會

地址：10041台北市中正區忠孝西路1段50號20樓之8

網址：http：//www.gsthome.com.tw

編輯顧問　陳慶餘　台灣老年醫學會/理事長
國家衛生研究院老年醫學研究組/主任

總編輯

李燕鳴　花蓮慈濟醫院家醫科主治醫師/副教授
盧豐華　國立成功大學附設醫院家庭醫學部主治醫師/副教授

編審委員

郭月霞　國立台灣大學附設醫院營養部/營養師
彭睿華　國立台灣大學附設醫院護理部/護理師
詹鼎正　國立台灣大學附設醫院老年醫學部/主治醫師
劉文俊　台灣老年醫學會/秘書長
賴秀昀　台北榮民總醫院家醫部/老年專科醫師
羅美芳　國立台灣大學護理學系所/助理教授
蘇富美　國立師範大學衛生教育學系所/副教授

協助撰寫

吳至行　國立成功大學附設醫院家庭醫學部主任/副教授
吳晉祥　國立成功大學附設醫院家庭醫學部主治醫師/副教授
黃盈祥　國立成功大學附設醫院家庭醫學部/主治醫師
陳人豪　國立台灣大學附設醫院內科部/主治醫師
陸鳳屏　國立台灣大學附設醫院內科部/主治醫師
傅振宗　花蓮慈濟醫院新陳代謝暨內分泌科主治醫師/副教授
黃麗卿　台北馬偕醫院家庭醫學科/主任
邱國樑　台灣睡眠醫學學會秘書長
顏啟華　台灣老年醫學會主任醫師
陳鈺如　台灣護理學督導
林怜利　經國管理暨健康學院主任
尚曉菁　彰化縣衛生局保健課長

99年度編修小組

王英偉　花蓮慈濟醫院家庭醫學科主治醫師/副教授
蔡娟秀　慈濟醫技術學院護理系/副教授
謝至鏗　花蓮慈濟醫院家庭醫學科/主治醫師

執行小組：行政院衛生署國民健康局 陳姿伶、周美珍、林倩玉

國家圖書館出版品預行編目(CIP)資料

健康老化 ： 銀髮族保健手冊 ／ 行政院衛生署國民健康局著. -- 初版. -- 臺北縣新莊市 ： 衛生署國民健康局, 民 99.12
面 ； 公分
ISBN 978-986-02-6511-8(精裝附光碟片)

1.老年醫學 2.中老年人保健 3.自我照護 4.老人養護

417.7 99025795

書　　名：健康老化銀髮族保健手冊
著(編、譯)者：行政院衛生署國民健康局
出版機關：行政院衛生署國民健康局
地　　址：40873 台中市南屯區干城街 95 號 4 樓
網　　址：http：//www.bhp.doh.gov.tw/
電　　話：04-22550177
出版年月：99 年 12 月
版(刷)次：初版 20,000 本
其他類型版本說明
本書同時登載於行政院衛生署國民健康局 健康九九網站，網址為
http：//health99.doh.gov.tw/
定價：50 元
展售處
台北　國家書坊台視總店
地址　10502 台北市松山區八德路 3 段 10 號 B1
電話　(02)2578-1515
台中　五南文化廣場
地址　40042 台中市中區中山路 6 號
電話　(04)2226-0330
GPN：
ISBN：
著作財產權人：行政院衛生署國民健康局
本書保留所有權利。欲利用本書全部或部分內容者，需徵求著作財產權人行政院衛生署國民健康局同意或書面授權。請洽行政院衛生署國民健康局(電話：04-22550177-535)
All rights reserved. Any forms of using or quotation, part or all should be authorized by copyright holder Bureau of Health Promotion, Department of Health, R.O.C..
Please contact with Bureau of Health Promotion. Department of Health, R.O.C.. (Tel：866-4-22550177)

行政院衛生署國民健康局
40873 台中市南屯區黎明路二段503號5樓
電話：04-22591999　傳真：04-22545510
網址：http：//www.bhp.doh.gov.tw/

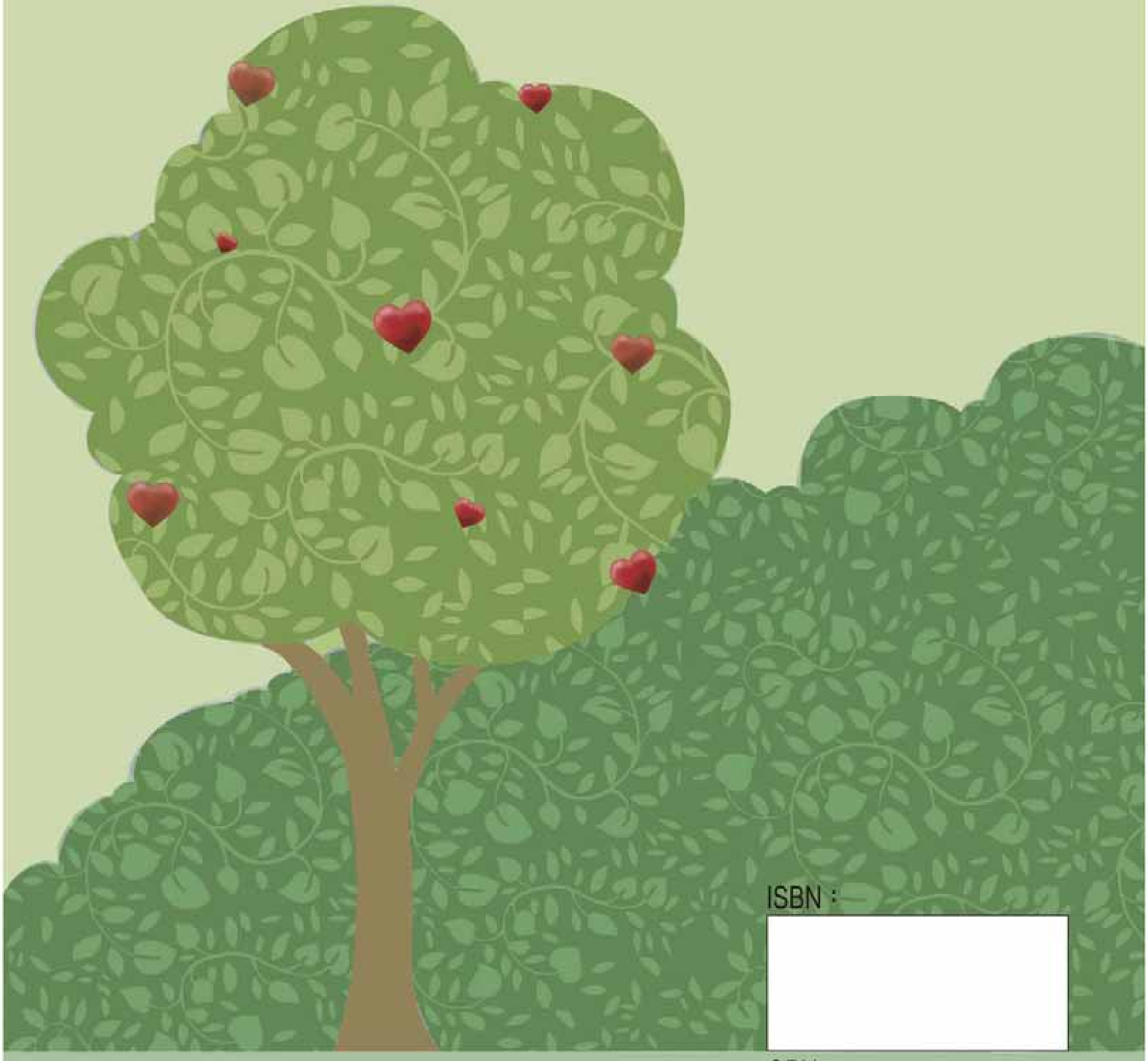

ISBN：

GPN：
定價:50元整

健康老化

DVD VIDEO

銀髮族保健

片　　長：25分鐘

主題內容：健康生活型態、自我健康管理、常見慢性病的自我管理、老年症候群、安居宜養、老人照護資訊等。

製作年份：99年

GPN：
ISBN：

監　製：
行政院衛生署國民健康局
電　　話：04-22591999

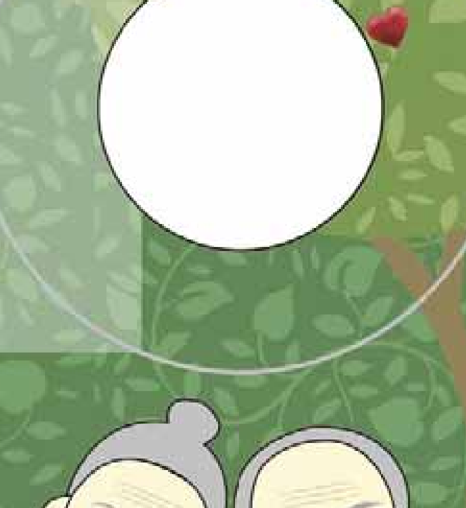

Supplement: Multimedia Appendix 6 [file jmir_v26i1e53450_app6.pdf]
