# Supplementary figures and images for: A Web-Based Resilience-Enhancing Program to Improve Resilience, Physical Activity, and Well-being in Geriatric Population: Randomized Controlled Trial
Source: J Med Internet Res. 2024 Jul 25;26:e53450. doi: 10.2196/53450 (PMC11310648; doi:10.2196/53450)

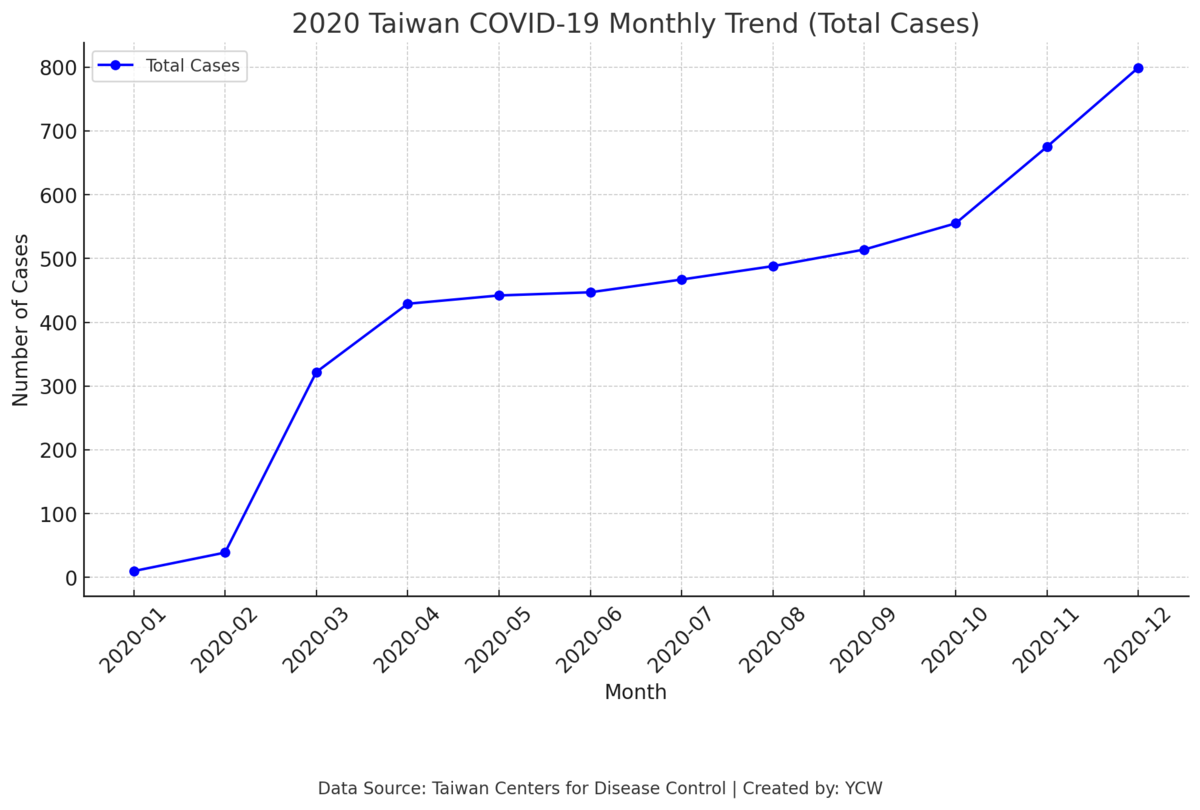

Supplement: Multimedia Appendix 7 [file jmir_v26i1e53450_app7.png]
