# Supplementary material for: A Web-Based Resilience-Enhancing Program to Improve Resilience, Physical Activity, and Well-being in Geriatric Population: Randomized Controlled Trial
Source: J Med Internet Res. 2024 Jul 25;26:e53450. doi: 10.2196/53450 (PMC11310648; doi:10.2196/53450)
Supplement: Multimedia Appendix 9 [file jmir_v26i1e53450_app9.pdf]

Multimedia Appendix 9

Table S1. Estimated marginal mean score of resilience over time

Outcomes		T0[a] intervention	T0[a] control	T1[b] intervention	T1[b] control	T2[c] intervention	T2[c] control
**Resilience, mean (SD)**		138.84 (22.51)	135.78 (30.09)	144.40 (18.80)	139.29 (27.61)	146.28 (18.60)	140.62 (29.05)
	**Self-reliance, mean (SD)**	33.29 (6.28)	32.59 (7.55)	34.39 (5.42)	33.69 (6.80)	35.39 (5.20)	34.70 (6.98)
	**Perseverance, mean (SD)**	43.00 (7.81)	42.91 (9.29)	46.01 (6.72)	43.29 (9.65)	46.00 (7.01)	43.30 (9.65)
	**Equanimity, mean (SD)**	33.19 (5.80)	32.78 (8.08)	34.14 (4.98)	33.36 (7.81)	34.66 (5.29)	33.42 (7.93)
	**Meaningful, mean (SD)**	17.52 (2.93)	16.22 (4.48)	18.00 (2.32)	17.10 (3.50)	18.01 (2.63)	17.03 (3.66)
	**Existential aloneness, mean (SD)**	11.84 (2.40)	11.28 (3.10)	11.82 (1.70)	11.94 (2.27)	12.35 (1.52)	11.85 (2.53)

[a] baseline
[b] 4-week after completed the resilience program
[c] 12-week after completed the resilience program.

Table S2 Estimated marginal mean score of physical activity over time

Outcomes		T0[a] intervention	T0[a] control	T1[b] intervention	T1[b] control	T2[c] intervention	T2[c] control
**Physical activity, mean (SD)**		162.16 (76.25)	111.75 (60.08)	166.16 (70.66)	127.68 (88.59)	150.01 (62.53)	122.75 (68.85)
	**Recreational activity, mean (SD)**	50.06 (44.10)	31.22 (32.43)	58.51 (46.39)	46.63 (50.85)	49.46 (41.25)	41.99 (41.28)
	**Household activity, mean (SD)**	86.25 (35.65)	66.00 (38.73)	85.14 (32.81)	66.98 (36.87)	80.66 (33.09)	74.19 (37.31)
	**Occupational activity, mean (SD)**	25.84 (30.76)	14.55 (29.66)	22.52 (24.17)	14.07 (24.71)	19.89 (24.61)	6.57 (10.71)

[a] baseline
[b] 4-week after completed the resilience program
[c] 12-week after completed the resilience program.

Table S3 Estimated marginal mean score of well-being over time

Outcomes		T0[a] intervention	T0[a] control	T1[b] intervention	T1[b] control	T2[c] intervention	T2[c] control
**Well-being, mean (SD)**		92.29 (12.69)	92.28 (17.15)	95.65 (11.09)	96.29 (17.38)	94.23 (10.73)	95.22 (15.63)
	**Life satisfaction, mean (SD)**	27.13 (3.90)	27.06 (5.54)	27.84 (3.61)	28.54 (5.32)	28.06 (3.82)	28.36 (4.51)
	**Interpersonal relationship, mean (SD)**	22.74 (3.54)	23.38 (5.13)	23.83 (3.19)	24.88 (4.81)	23.88 (3.35)	24.22 (4.16)
	**Self-assurance, mean (SD)**	23.94 (3.23)	22.88 (4.11)	23.62 (2.55)	23.38 (4.15)	24.14 (2.87)	23.53 (4.57)
	**Physical and moral integrity, mean (SD)**	18.48 (3.19)	18.97 (4.48)	19.82 (2.78)	19.60 (4.69)	18.81 (2.87)	19.07 (4.27)

[a] baseline

[b] 4-week after completed the resilience program

[c] 12-week after completed the resilience program.
